# The Relationships, Employment, Autonomy, and Life Satisfaction (REALS) Measures for Autistic Adults and Adults With Other Intellectual and Developmental Disabilities: Psychometric Testing of the Self‐Report and Proxy Versions

**DOI:** 10.1002/aur.70002

**Published:** 2025-02-12

**Authors:** Caitlin M. Conner, Lan Yu, Kristen T. MacKenzie, Katharine N. Zeglen, Elizabeth L. Rutenberg, Paul A. Pilkonis, Shaun M. Eack, Carla A. Mazefsky

**Affiliations:** ^1^ Department of Psychiatry University of Pittsburgh School of Medicine Pittsburgh Pennsylvania USA; ^2^ Center for Research on Health Care University of Pittsburgh School of Medicine Pittsburgh Pennsylvania USA; ^3^ Department of Psychology University of Pittsburgh Pittsburgh Pennsylvania USA; ^4^ Department of Social Work University of Pittsburgh Pittsburgh Pennsylvania USA

**Keywords:** adult outcomes, autism, intellectual and developmental disabilities, measurement

## Abstract

Research and clinical work demonstrate that adults with intellectual and developmental disabilities (IDDs; including autistic adults and adults with other IDDs) struggle with key outcomes in adult life, including social relationships, employment, autonomy, and life satisfaction. However, few validated measures exist to measure these outcomes in adults with IDDs. The Relationships, Employment, Autonomy, and Life Satisfaction (REALS) Measures were created using methods developed by the Patient‐Reported Outcomes Measurement Information System (PROMIS) to assess these outcomes. Large item pools were generated for the four domains, and, in field testing, 875 adults with IDDs (90% autistic; 18.4% with intellectual disability or a non‐autism IDD) and 911 proxy reporters (caregivers; 79% autistic; 48.3% with intellectual disability or a non‐autism IDD) completed 108 and 74 items, respectively, using response options capturing frequency, level of support needed, and satisfaction. The structure and item content of the REALS Measures were determined through an iterative process using both classical test theory and item response theory analyses. The final versions include 19 self‐report and 14 proxy‐report measures, with a range of 3 to 14 items each. The measures have excellent psychometric properties, high precision, and acceptable respondent burden. Thus, they are applicable for service provision, clinical, and research arenas for autistic adults and adults with other IDDs, though additional testing in IDD is warranted and evidence supporting self‐report use in IDD is more limited.

**Summary**

- This paper introduces a group of new measures called the REALS (Relationships, Employment, Autonomy, and Life Satisfaction) Measures.
- Eight hundred and seventy‐five adults with intellectual and developmental disabilities (IDDs) and 911 caregivers of adults with IDDs completed items about the person's social activities, work and school activities, daily life tasks, and life satisfaction.
- Advanced statistical techniques were applied to identify the best final items.
- The REALS Measures consist of 19 short self‐report measures and 16 short proxy‐report measures that can be used in clinical and research settings to measure the status of adults with IDDs in important life domains and to assess their progress towards an improved quality of life.

## 1 Introduction

Increasing attention has been focused on two critical gaps in research with adults with intellectual and developmental disabilities (IDDs; including autistic adults and those with other IDDs) – (1) the need to better measure important outcomes in adulthood (Brugha et al. 2015; Esbensen et al. 2010; Nicolaidis et al. 2020; Shattuck et al. 2020; Shogren et al. 2021; Walton et al. 2022), and (2) the need for measures that are sensitive to change, especially in the context of treatment or other active interventions. Many commonly used outcome measures are problematic for use with adults with IDDs, including measures initially developed for children with IDDs or for other clinical populations (Berry‐Kravis et al. 2013; Esbensen et al. 2017; Hart et al. 2017; Henninger and Taylor 2013; Koslowski et al. 2016; Nicolaidis et al. 2020). Therefore, options for measuring important aspects of adult life are limited for autistic adults and those with other IDDs (Brugha et al. 2015; Wigham et al. 2020; Fujiura 2012; Shogren et al. 2021), including assessment of social relationships, employment, and autonomy (Benevides et al. 2020; Benevides and Cassidy 2020; Brugha et al. 2015; Kramer et al. 2019; Raymaker et al. 2022).

Due to the lack of available measures, researchers have often resorted to reporting on adult outcomes using global categories or labels (e.g., poor, good, and very good outcome). Analyzing outcomes using broad categories, however, obscures more nuanced information about adult life and provides little sensitivity to change for longitudinal studies or interventions, which limits clinical utility (Henninger and Taylor 2013; Rix 2023). Furthermore, it is well documented that the use of broad categories inaccurately characterizes support needs (Henninger and Taylor 2013; Kapp 2023; Whiteley, Carr, and Shattock 2021; Chapman and Veit 2020; Chapple and Worsley 2021; Rix 2023). Measuring adult life dynamically, across multiple domains, is more optimal to assess support needs and reflect the complexity of adult life precisely and respectfully.

Currently, the Vineland Adaptive Behavior Measures −3 (VABS‐3; Sparrow, Cicchetti, and Saulnier 2016) is the gold‐standard assessment of global functioning in IDD (Hamburg et al. 2019). It remains a required measure for participants of all ages, including adults, for important IDD research initiatives, such as NIH Autism Center of Excellence grants and the Simons Powering Autism Research (SPARK) program. Although the VABS‐3 provides norms up to age 90, it was initially developed for children up to age 18 for the purposes of qualifying for services or to inform individualized education plans. However, the VABS‐3 has complex basal/ceiling‐based scoring, and it requires proxy report. The Adaptive Behavior Assessment System, Third Edition (ABAS‐3; Harrison and Oakland 2015) is a similar questionnaire that assesses adaptive behaviors in individuals from age 0–89 years. Unlike the VABS‐3, the ABAS‐3 has separate versions for ages 0–5, 5–21, and 16–89 years, separate versions and norms for caregivers and teachers, and a self‐report version for 16–89 year‐olds. The VABS‐3 overlooks a number of important aspects of adult life, such as any work‐related items, and both the VABS‐3 and ABAS‐3 lack items related to work readiness. Neither measure accounts for the level of support that a person needs in completing tasks, instead the measures instruct the rater to respond based on what a person is able to do independently. The VABS‐3 response options refer to how frequently the person does a task (Never, Sometimes, or Usually), and the ABAS‐3 response options assess the frequency completing a behavior without support (0 = ‘is not able to do behavior,’ 1 = ‘never (or almost never),’ 2 = ‘sometimes,’ 3 = ‘always (or almost always)’, with an option to check a box if you guessed). Often reporters are unsure of how to respond to items if the person only performs that behavior when another person provides a level of support. In addition, neither measure is freely available, scoring is complex, and it requires proxy report. These measures of adaptive skills were also not designed to be sensitive to change. Finally, traditional adaptive behavior scales tend to focus on discrete skills and we intended to develop a measure to better understand outcomes (i.e., not one's social skills, but their actual social activities and relationships). Perhaps most importantly to a person completing the measure, both the ABAS‐3 and VABS‐3 are lengthy questionnaires and were not designed to administer one topic at a time. Other options, such as the Waisman Activities of Daily Living Scale (Maenner et al. 2013), cover some content areas, but other areas do not have similar measure options, which means that short, targeted, change‐sensitive measures are lacking for interventions.

Fortunately, there has been a recent increase in efforts to improve and increase options for adult questionnaires. This is due to growing appreciation that the psychometric properties of measures that were not developed for or tested in IDD can be problematic, as well as recognition of the need to ensure outcome measures capture the areas of adult life that adults with IDD feel are important. Examples of some of these recent efforts include the AASPIRE Patient‐Reported Outcomes Project (Nicolaidis et al. 2020), development of a self‐report version of the Emotion Dysregulation Inventory (Mazefsky et al. 2024), and development of a measure of suicidality for autistic adults (Conner et al. 2023). The intention was not to duplicate these efforts, but to complement them by focusing on developing item banks for relationships, employment, and autonomy, areas that the IDD community has identified as priorities (Benevides et al. 2020; Benevides and Cassidy 2020; Kramer et al. 2019; Raymaker et al. 2022).

To fill these gaps in the literature, we developed the REALS (Relationships, Employment, Autonomy, and Life Satisfaction) Measures. The item development process is described in detail in MacKenzie et al. (2024) and based on PROMIS guidelines (PROMIS 2013). In brief, an item pool was produced after a comprehensive literature review was completed and a conceptual model consisting of relationships, employment, and autonomy was created. The conceptual model's focus on relationships, employment, and autonomy was based on priorities stated by adults with IDDs in prior research (Benevides et al. 2020; Benevides and Cassidy 2020; Kramer et al. 2019; Raymaker et al. 2022). Additionally, we believed that it would be useful to have satisfaction scales that mirror the other conceptual domains (i.e., satisfaction with autonomy). Other measures frequently used in the literature for treatment trials, such as the Aberrant Behavior Checklist (Aman et al. 1985) are sensitive to change, but do not cover the same content area (such as social, employment, and independent living domains) The conceptual model, based on PROMIS guidelines, contained domains (adult life), subdomains (relationships, autonomy, employment), factors, and facets. Multiple items were drafted to ensure coverage across all facets of the domains. The conceptual model was also reviewed by experts (IDD and autism researchers, measure development researchers, service providers, and parent/caregivers) and then refined following input from adults with IDD during cognitive interviews.

Cognitive interviews were completed with 15 autistic adults, 7 adults with other IDDs, 13 caregivers of autistic adults, and 10 caregivers of adults with IDDs (see MacKenzie et al. 2024, for more information). Cognitive interviews included review of the initial item pool and probed understanding of items, response options, and instructions, as well as decision‐making processes when selecting a response. This feedback, discussed by the research team and shared with consultants, was used to revise the measure. Some of the main areas of feedback included: (a) altering and reducing the response options to simplify and to enhance practice relevance, (b) to simplify and to enhance practice relevance, removing highly temporal items that did not fit into a three‐month timeframe such as voting or going on vacation, and (c) separating employment‐related items into two groups based on current employment (i.e., work performance‐based items for individuals currently in work or school, and work readiness‐based items for individuals not currently working). Additionally, proxy reporters felt that they were unable to genuinely and reliably rate satisfaction for their adult children and therefore the satisfaction items were removed from proxy report versions.

Revisions were then made, including (a) changes to individual items, (b) development of two sets of response options (frequency and the amount of support one needs, in addition to retaining self‐report satisfaction), and (c) removal of satisfaction from the proxy report. Next, a second round of cognitive interviews was conducted with a subsample of participants (*n* = 3), with positive feedback. We also removed items based on suggestions that some items should only be rated for frequency and not support, romance‐related items were removed from proxy report, and then, the REALS measures item bank was finalized for psychometric data collection.

The purpose of this manuscript is to provide the results of psychometric testing of the REALS Measures, following the edits and revisions described above and in MacKenzie et al. (2024). The aim in psychometrics was to develop a suite of brief, efficient, focused measures reflecting aspects of relationships, employment, autonomy, and satisfaction for autistic adults and adults with other IDDs. Therefore, we tested the factor structure and evaluated item properties to identify the most precise and informative items, in addition to evaluation of convergent and divergent validity.

## 2 Methods

### 2.1 Participants

Participants included autistic adults, adults with other IDDs, as well as proxy reporters for autistic adults and those with other IDDs, for example, parents, adult siblings, and other relatives (see Table 1 for descriptive characteristics of the sample). An autism sample was collected through the Simons Powering Autism Research (SPARK) registry. Inclusion in the SPARK registry requires a self‐reported community diagnosis of an autism spectrum disorder. Previous research to verify the validity of diagnoses in the SPARK sample found that 98.8% of a SPARK subsample had confirmed ASD diagnoses via medical records (Fombonne et al. 2022). SPARK identified its autistic self‐reporters aged 18+ in two ways: Dependent Adults, who were registered by and with caregivers; and Independent Adults, who joined the registry themselves and were given the option to invite an Outside Reporter (i.e., non‐registry member and adult who knew the Independent Adult well) to participate. In addition to SPARK, over 160 local and national autism and IDD organizations were contacted during recruitment; these organizations were the sources of the other IDDs respondents. In total, 875 adults (788 identified as autistic) and 911 caregivers (724 caregivers of autistic adults) completed the study (see Table 1). All self‐reporters and proxy reporters were combined across those with autism and IDD for analyses. In Table 1, we also display statistics for those who have autism without intellectual disability or another neurodevelopmental condition that is part of the IDD umbrella versus those with intellectual disability or a reported IDD (may also have co‐occurring autism). While 18.4% of the self‐report sample included those with reported ID or an IDD, 48.3% of the proxy report included those with ID or an IDD.

**TABLE 1:** Demographic information.

	Self‐report (*n* = 875)	Proxy‐report of adult (*n* = 911)	Caregiver (*n* = 911)
	*N* (%)	*N* (%)	*N* (%)
**IDD status**			Not asked
IDD	161 (18.40)	440 (48.3)	
Autism only	714 (81.60)	471 (51.70)	
**Type of IDD**			Not asked
**Autism**	794 (90.74)	728 (79.91)	
Autism and any IDD	80	257	
Autism and Intellectual Disability (ID)	53	178	
Autism and Down Syndrome	0	8	
Autism and Fragile X	0	32	
Autism and Williams Syndrome	0	1	
Autism and another IDD diagnosis	27	38	
Autism only	714	471	
**Not autism**	81 (9.26)	183 (20.01)	
Down syndrome	14	85	
Fragile X	27	46	
Intellectual disability	25	12	
Williams syndrome	6	10	
Other	27 (including Prader Willi = 1, Misc (ADHD, depression, anxiety) = 26)	31 (including Prader Willi = 10, Angelman Syndrome = 4, Misc (ADHD, dementia, other mental health conditions) =17)	
**Verbal ability**			Not Asked
Minimally verbal	42 (4.80)	160 (17.60)	
**IQ score**			Not Asked
IQ less than 70	40 (4.60)	155 (17.00)	
**Age (years)**	**M (SD)** 35.93 (12.89); range 18–77	**M (SD)** 29.36 (9.87); range 18–71 (*N* = 668)	Not Asked
**Race**	***N* (%)**	***N* (%)**	***N* (%)**
Asian/Pacific Islander	26 (3.00)	32 (3.50)	22 (2.40)
Black	64 (7.40)	54 (5.90)	43 (4.70)
Native American	42 (4.80)	22 (2.40)	20 (2.20)
Hispanic/Latine	48 (5.50)	58 (6.40)	51 (4.30)
White	682 (77.90)	732 (80.40)	761 (83.50)
Other	13 (1.50)	13 (1.40)	14 (1.54)
**Gender**			Not asked
Male	347 (40.0)	581 (63.80)	(programming error)
Female	417 (48.0)	296 (32.50)	
Nonbinary/Gender fluid	67 (7.70)	23 (2.50)	
Agender/No gender	6 (0.70)	0	
Unsure/questioning	20 (2.30)	1 (0.10)	
Other/Prefer not to answer	4 (0.50)	5 (0.50)	
Missing	14 (1.60)	5 (0.50)	
**Marital status**			
Single, not actively dating	436 (50.20)	702 (77.10)	93 (10.20)
Dating not in a committed relationship	63 (7.30)	45 (4.90)	12 (1.30)
In committed relationship/married	280 (32.30)	130 (14.30)	634 (69.60)
Divorced/separated	61 (7.0)	14 (1.50)	123 (13.40)
Widow	10 (1.20)	3 (0.30)	35 (3.80)
Prefer not to answer/Missing	25 (2.86)	17 (1.90)	14 (1.54)
**Education**			
Less than 8th grade	7 (0.80)	44 (4.80)	2 (0.20)
Some high school	40 (4.60)	90 (9.90)	11 (1.20)
Finished high school or equivalent	189 (21.80)	472 (51.80)	80 (8.80)
Some college	226 (26.0)	156 (17.10)	282 (31.0)
Associate's degree or technical school	246 (28.30)	82 (9.0)	273 (30.0)
Bachelor's degree (BA, BS)	154 (17.70)	49 (5.40)	256 (28.10)
Post graduate degree	6 (0.70)	13 (1.40)	1 (0.10)
Missing	7 (0.80)	5 (0.50)	6 (0.70)
**Income**			
Less than $20.999	428 (49.30)	672 (73.80)	163 (17.90)
$21,000 to $35.999	131 (15.10)	76 (8.30)	129 (14.20)
$36,000 to $50.999	86 (9.90)	23 (2.50)	119 (13.10)
$51,000 to $65.999	47 (5.40)	10 (1.10)	100 (11.0)
$66,000 to $80.999	33 (3.8)	16 (1.80)	65 (7.10)
$81,000 to $100.999	31 (3.60)	8 (0.90)	77 (8.50)
$101,000 to $130.999	23 (2.60)	6 (0.70)	55 (6.0)
$131,000 to $160.999	11 (1.30)	0	36 (4.0)
Over $160,000	8 (0.90)	2 (0.20)	55 (6.0)
Missing/Prefer not to answer	77 (8.80)	98 (10.70)	112 (12.20)
**Employment**			
Working full time, paid	282 (32.50)	129 (14.20)	410 (45.0)
Working part time, paid	212 (24.40)	206 (22.60)	183 (20.10)
Unpaid student intern	12 (1.4)	33 (3.60)	2 (0.20)
Not working at this time	161 (18.50)	184 (20.20)	64 (7.0)
Unable to work	176 (20.30)	345 (27.90)	90 (9.90)
Retired	24 (2.80)	7 (0.80)	153 (16.80)
Missing	8 (0.91)	7 (0.80)	9 (1.0)
**Housing arrangement**			Not Asked
Alone	177 (20.20)	61 (6.70)	
With roommate or romantic partner	277 (31.70)	137 (15.0)	
With parents or other relatives	357 (40.80)	617 (67.70)	
In a residential facility	2 (0.20)	15 (1.60)	
In a group home	13 (1.50)	48 (5.30)	
Homeless	6 (0.70)	2 (0.20)	
Other	43 (4.90)	31 (3.40)	

*Note*: Proxy‐report of adult information is concerning the subject of their report (for example, the adult child of the parent who is reporting).

Abbreviation: IDD = Intellectual or Developmental Disability.

Table 1 summarizes the demographic characteristics of the self‐and proxy‐report participants. The majority of self‐report participants were White, autistic, and had fluent speech. The majority of proxy‐report participants were White, in a committed relationship or married, and completed post‐secondary education. The person about whom proxy‐reporters reported were majority White, male, autistic, and had fluent speech (although a smaller percentage than the self‐report sample).

### 2.2 Measures

#### 2.2.1 Demographic and Background Information

Each participant was administered a questionnaire documenting sex, gender, age, education level and history, employment, housing arrangements, IDD diagnoses, and preferred autism language (as relevant). Proxies completed an item about their relationship to the person they were rating. Information about whether or not they were in work or school was used to determine if they received the Work/School Performance items (if currently in work or school) or Work Readiness (if neither attending school nor working). Race and ethnicity were collected as mandated by the US National Institutes of Health, with the ability to select more than one racial identity. For analysis, we used a deterministic bridging method to assign multi‐racial participants to the single race they identified with that had the highest prevalence in the sample other than White (thus prioritizing their minoritized racial identity). This resulted in racial categories of Asian American and Pacific Islander, Black or African American, Hispanic/Latine, Native American, White, or Other.

#### 2.2.2 Relationships, Employment, Autonomy, and Life Satisfaction Measures (REALS) Measures

For psychometric analyses, the REALS Measures included 108 items for self‐report and 74 items for proxy‐report. Items were based on the conceptual model and fell into four separate domains: social relationships (20 items for self‐report and 20 for proxy report), employment and school (13 items for self‐report and 13 for proxy report), autonomy (41 items for self‐report and 41 for proxy report), and satisfaction in the three domains (34 items—self‐report only). There were separate versions with different response options, which were taken from or adapted from previous PROMIS measures (see MacKenzie et al. 2024, for more information). One version of the measures included a five‐point frequency rating (never, rarely, sometimes, often, and almost always). Another version included a five‐point degree of support needed (unable to do even with support, needed a lot of support, needed some support, needed a little support, was able to do without any support). There were some exceptions for items that were only rated by frequency when ratings for support needed were inappropriate (e.g., romance and sexual intimacy items). Finally, for self‐report only, there were additional items rated based on a four‐point satisfaction scale (not at all, a little bit, quite a bit, completely). The final REALS Measures and scoring tables are available for free via a request form at www.reaact.pitt.edu. Scoring involves summing raw scores and converting to a t‐score or theta score with a conversion table.

#### 2.2.3 Measures for Validity Correlations

Measures of activities of daily living, adaptive behavior, and quality of life were administered to examine convergence of the REALS Measures with other established measures of adult and IDD outcomes.

Waisman Activities of Daily Living Scale (W‐ADL; Maenner et al. 2013). Activities of daily living were assessed using the W‐ADL, which is a 17‐item measure assessing instrumental daily living activities (e.g., washing/bathing). While initially designed as a caregiver‐report measure, both self‐ and proxy‐report versions were completed in this study. Items are rated on a 3‐point scale, with higher scores reflecting greater independence in performing daily living activities.

Specific Level of Functioning Scale (SLOF; Schneider and Struening 1983). The SLOF is a 30‐item proxy report measure of functional outcomes that assesses functioning across interpersonal relationships, social acceptability, community activities, and work skills. Items are rated on a 5‐point scale, with higher scores indicated better functional outcomes. The social acceptability subscale was viewed as inappropriate for this population and not included in analysis. Similar to the W‐ADL, the SLOF is traditionally a proxy report measure. However, adults were also asked to provide self‐report responses for each measure in this study.

Vineland Adaptive Behavior Scales, Third Edition (VABS: Sparrow, Cicchetti, and Saulnier 2016). The VABS is a 502‐item proxy report measure of adaptive behavior that has been validated and widely used in IDD research. Items are rated on a 3‐point frequency scale with summary and composite scores provided for multiple domains, and convergent validity analyses focused on the adaptive behavioral composite, along with daily living skills, communication, and socialization summary scores. Only proxy‐reporters completed the VABS for this study.

World Health Organization Quality of Life Assessment‐Brief (WHOQOL‐BREF; World Health Organization 2004). The WHOQOL‐BREF is a 26‐item self‐report questionnaire of quality of life that has been validated in prior research on adults with IDDs. Each item is rated on a 5‐point Likert scale, where higher scores are higher quality of life. The WHOQOL‐BREF contains an overall health satisfaction item, a 7‐item physical health scale, 6‐item psychological health scale, 3‐item social relationship scale, and an 8‐item environment scale.

### 2.3 Procedures

#### 2.3.1 Ethics and Consent

All procedures performed in studies involving human participants were in accordance with the ethical standards of the University of Pittsburgh Institutional Review Board (Study Number: 19080355) and with the 1964 Helsinki declaration and its later amendments or comparable ethical standards. No animal subjects were involved in this study. Data were collected between May 2, 2022, and February 1, 2023.

#### 2.3.2 Recruitment

All participants provided online informed consent and, where applicable, assent (for those Dependent Adults whose legal guardians first provided consent), and all participants completed the survey battery online. SPARK directly contacted eligible adults in their registry via email to invite their participation in the study. Other listservs and research registries, including Down Syndrome Connect through the National Institutes of Child Health and Human Development, the Global Prader‐Willi Syndrome Registry, the Williams Syndrome Registry, and the University of North Carolina's Carolina Institute for Developmental Disabilities' Fragile X Registry, also emailed or posted information regarding the study.

#### 2.3.3 Online Study Completion

To minimize the possibility of fraudulent survey responses, the research team used several best practices for conducting online research studies: requiring potential participants to email in order to receive the study link, triangulation procedures to confirm the identities of referrals from enrolled participants, real‐time screening via a method of the participant's choice (e.g., phone call or non‐verbal secure video call with chat), online tools designed to eliminate bots (e.g., “honeypots”, which are decoy systems or servers that look like a legitimate target to lure bots and bad actors away from real assets), enrollment monitoring, regular data audits during data collection, and random post‐survey feedback calls to participants. Participants recruited via the SPARK registry were pre‐screened by SPARK's secure platform according to personal information in their private registry profiles, completed upon joining the registry and updated regularly; study staff did not have direct access to these individuals per SPARK's privacy agreement. Participants recruited from other registries as well as local and national organizations were screened in real‐time via the methods previously described; these individuals learned about the study via targeted emails and e‐newsletters from familiar organizations, personal referrals from friends and healthcare professionals with whom we shared the study electronic flyer. Interested individuals contacted study staff via phone or email provided on the flyer and answered screening questions to determine if they were eligible. Enrollment was monitored by study staff on a daily basis via the secure database by looking at survey battery completion times (project staff tested during piloting and determined a low threshold for humans clicking through the surveys as quickly as possible), contact information, mismatched names and email addresses, and other oddities such as misspellings, the use of all capital letters, and suspicious email addresses (i.e., what look like a collection of random numbers and characters). Post‐survey feedback calls and emails were administered randomly (IDs chosen via a random number generator) to roughly 10% of the total who completed surveys as well as any individual whose participation raised a red flag to study staff (e.g., exceptionally low completion time for survey battery, first and last names entered for both first and last name fields).

### 2.4 Analyses

#### 2.4.1 Factor Analysis

Factor analyses were conducted using Mplus 6.2 with promax rotation (Muthén and Muthén 1998). We first conducted analyses with the self‐report items, followed by proxy‐report items. All analyses were done separately for each response option. The sample was randomly split for exploratory factor analysis (EFA; *n* self‐report = 438; *n* proxy report = 456) and confirmatory factor analysis (CFA; *n* self‐report = 437; *n* proxy report =455). Once the best fitting EFA solution was determined, CFA was run to confirm the structure.

We examined factor loadings, scree plots, and eigenvalues in the EFAs, with a focus on the ratios of eigenvalues in EFAs and the relative proportions of variance accounted for by the factors. Individual items were dropped because of factor loadings less than 0.45, cross‐loadings between factors, and clinical judgment regarding content validity. In two cases, items with factor loadings just below threshold (e.g., 0.42) were retained for consistency across frequency and support response options; (see Table S4). There is no standard cut‐off for factor loadings though 0.3 is commonly used, making our approach more conservative. Decisions were also guided by our goal to develop brief, focused scales, and, as much as possible, to maintain consistency between frequency and support ratings and between self‐ and proxy‐report.

#### 2.4.2 Item Response Theory (IRT) Analysis

After completing EFA and CFA, we evaluated the REALS item‐level properties. The most commonly used IRT model for polytomous items (i.e., items with three or more ordinal response categories) is the two‐parameter graded response model (GRM; Samejima 1969). The GRM has a slope parameter and *n*−1 threshold parameters for each item, where *n* is the number of response categories. The slope parameter measures item discrimination, that is, how well the item differentiates higher versus lower levels of severity (or *Ɵ* in IRT terms). Useful items have larger slope parameters. Threshold parameters measure item difficulty, that is, the ease versus difficulty of endorsing different response options for an item. For example, the first threshold parameter for an item tells us where along the Ɵ scale of severity a respondent is more likely to endorse a response of “rarely” rather than “never.” Local dependency (LD) marginal chi‐square analyses identified redundant items due to high LD (residual correlations) with other items after controlling for their Ɵ levels, and one item of each LD pair was removed (with rare exceptions when the content of both items was judged to be clinically valuable). When making final decisions, we also emphasized the fit and item information values reflected in IRT models, below.

#### 2.4.3 Differential Item Functioning (DIF) Analysis

DIF occurs when participant characteristics such as sex or age affect measurement. DIF analyses flag an item if it is more or less difficult to endorse or more or less discriminating for different subgroups after controlling for comparable Ɵ levels. We conducted DIF analyses for age using a median split (< vs. > = 31.7 years) and sex assigned at birth, as we wanted to ensure satisfactory item performance across adult age and sex. For proxy report only, we also ran an analysis comparing those with autism “only” (*n* = 471) (here meaning without intellectual disability or an identified additional IDD) versus those with intellectual disability or IDD, with or without co‐occurring autism (*n* = 440). For DIF analyses, we used the Wald tests (Lord 1977) with the improved Supplemental EM algorithm (Cai 2008) embedded in IRTPRO 3.1 (Cai, Du Toit, and Thissen 2012). Items with significant DIF (*p* < 0.01) received further review for potential elimination.

#### 2.4.4 Additional Validity and Reliability Analyses

After all final items had been selected, we ran Pearson's correlations between the REALS Measures and the measures described above. We also calculated mean and SD scores for each group based on gender, autism only versus IDD, employed or not, and age (18–27, 28–40, and 41–78) as a supplemental analysis (Table S1). We also calculated Cronbach's alpha internal consistencies for each scale.

## 3 Results

### 3.1 Self‐Report

Although it was not our goal to develop an overall measure of adult outcome and we did not anticipate a single factor emerging because of the breadth of content in the initial item pools, we began with a EFA for all items within each response set separately to confirm our expectation. We therefore ran two initial EFAs with 1‐ through 10‐factor solutions generated for (1) all 74 items rated by frequency and (2) all 68 items rated for degree of support. Examination of the factor structures, scree plots, magnitude of eigenvalues, and fit indices did not reveal compelling factor models for the undifferentiated item pools (as expected).

**FIGURE 1:**
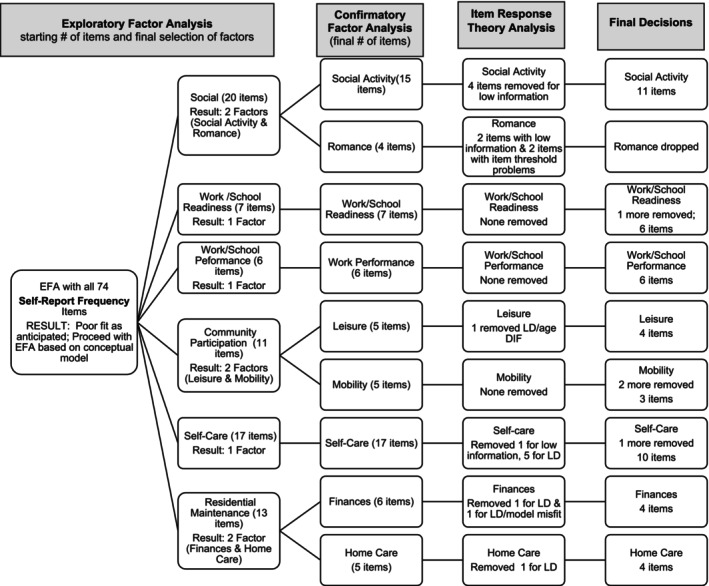
Self‐report frequency ratings—Summary of factor analysis and IRT decisions. LD = local dependency.

**FIGURE 2:**
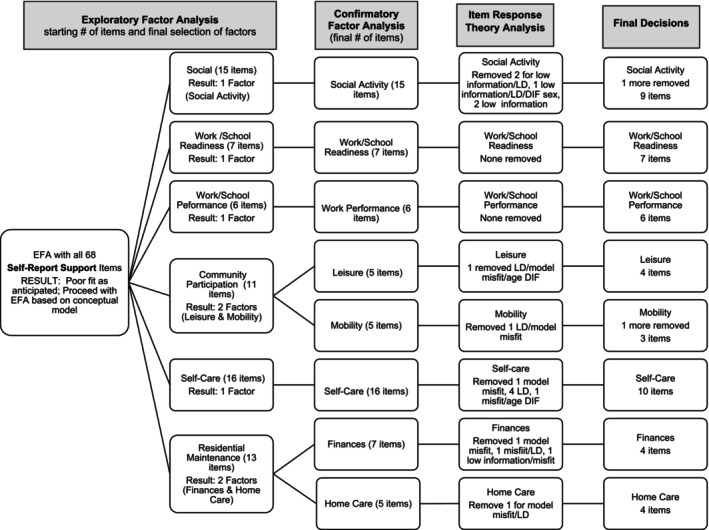
Self‐report support ratings—Summary of factor analysis and IRT decisions. LD = local dependency.

Therefore, as shown in Figures 1 and 2, we organized the frequency and support items into the content domains from our conceptual model. In keeping with our desire for brief, focused measures, we selected the “factor” level of our conceptual model as a starting point (MacKenzie et al. 2024) (i.e., the conceptual model includes a domain—adult life, subdomains—social, work/school, autonomy, then factors which we based EFA on, followed by facets). The only exception to this is that we conducted EFA on social relationships (vs. factors from our model of peer relationships and romantic relationships) because we did not have romance items on the version with “support needed” response options and we aimed to follow the same process across versions. Therefore, EFAs were run separately for items designed to tap social relationships, work/school readiness, work/school performance, community participation, self‐care, and residential maintenance. Separate EFAs for frequency and support items were conducted with 1‐ to 3‐factor solutions generated. Finally, an EFA was run with all satisfaction items in one model.

These analyses based on the conceptual model yielded acceptable factor models and fit statistics (see Table 2). Factor loadings from the final EFA and CFA models are presented in Tables S3–S9. Figures 1 (frequency ratings) and 2 (support ratings) include the flow of analyses and item winnowing including the initial number of items included and the number of factors and items following CFA. Factor analyses yielded 20 factors. Within social relationships frequency ratings, there were two factors that covered Social Activities (all CFA loadings > 0.54) and Romance (all CFA loadings > 0.70), correlated 0.50 with each other (a large effect size when using the heuristic of small = 0.1, medium = 0.3, large = 0.5). Within social relationships support ratings, there was a single factor of Social Activity items (all CFA loadings > 0.67). In the employment domain, there was strong support for a single factor for Work/School Readiness frequency items (all CFA loadings > 0.84) as well as for Work/School Readiness support items (all CFA loadings > 0.84). Results were similar for Work/School Performance, with a single factor for frequency (all CFA loadings > 0.62) and a single factor for support (all CFA loadings > 0.73). Within the Autonomy domain, there were five factors for frequency ratings and five factors for support ratings. Community participation yielded factors for Leisure (all CFA loadings > 0.56) and Mobility (all CFA loadings > 0.73) for Frequency, and Leisure (all CFA loadings > 0.80) and Mobility for Support (all CFA loadings > 0.69). Leisure and frequency were correlated 0.56 (large effect size) for frequency and 0.84 (large effect size) for support. Self‐care yielded a single factor for frequency (all CFA loadings > 0.53) and for support (all CFA loadings > 0.63). Finally, Residential Maintenance yielded factors for Finances (all CFA loadings > 0.74) and Home Care (all CFA loadings > 0.77) for Frequency, and Finances (all CFA loadings > 0.80) and Home Care for Support (all CFA loadings > 0.67). Leisure and frequency were correlated 0.61 for frequency and 0.76 for support (both large effect sizes).

**TABLE 2:** REALS measures CFA results.

Measure	REALS self‐report									
	Frequency response option					Support response option				
	No. of items	CLI	TLI	SRMR	RMSEA	No. of items	CLI	TLI	SRMR	RMSEA
Social activity (2 factor) – Social activities – Romance	19	0.905	0.892	0.074	0.119	15	0.956	0.949	0.046	0.118
Work readiness (1 factor)	7	0.996	0.995	0.033	0.116	7	0.993	0.989	0.027	0.181
Work/School performance (1 factor)	6	0.966	0.944	0.034	0.110	6	0.996	0.993	0.018	0.060
Autonomy‐Residential (2 factor) – Finances – Home care	11	0.933	0.914	0.060	0.141	12	0.951	0.939	0.055	0.146
Autonomy‐Participation (2 factor) – Leisure – Mobility	11	0.874	0.833	0.081	0.147	11	0.957	0.943	0.057	0.151
Autonomy‐Self Care (1 factor)	11	0.874	0.843	0.068	0.157	16	0.880	0.861	0.092	0.188
Satisfaction (3 factor) – w/Social activity – w/Work/School – w/Autonomy	27	0.912	0.903	0.073	0.105					

Measure	REALS proxy‐report^a^									
	Frequency response option					Support response option				
	No. of items	CLI	TLI	SRMR	RMSEA	No. of items	CLI	TLI	SRMR	RMSEA
Social activity (1 factor)	15	0.907	0.892	0.074	0.151	14	0.960	0.953	0.050	0.144
Work readiness (1 factor)	7	0.993	0.989	0.040	0.153	7	0.994	0.991	0.024	0.163
Work/School performance (1 factor)	6	0.976	0.961	0.037	0.115	6	0.995	0.991	0.016	0.099
Autonomy‐Residential (2 factor) – Finances – Home Care	10	0.971	0.962	0.059	0.127	10	0.977	0.969	0.048	0.144
Autonomy‐Participation (2 factor) – Leisure – Mobility	8	0.967	0.952	0.047	0.101	8	0.982	0.974	0.035	0.121
Autonomy‐Self Care (2 factor) – Self‐care – Sleep, diet, and exercise	15	0.863	0.838	0.106	0.149	15	0.942	0.931	0.071	0.156

^a^ Life satisfaction not included in REALS proxy‐report.

For satisfaction (Table S9), four items were removed prior to factor analysis to maintain consistency with content coverage in the frequency and support rating options of REALS Measures. An exploratory factor analysis with all 30 items yielded an optimal fit and interpretable model with items loading into three factors: Social, Work/School, and Autonomy. A three‐factor CFA confirmed these results, yielding a 7‐item Social Satisfaction factor (all CFA loadings > 0.84), 15‐item Autonomy Satisfaction factor (all CFA loadings > 0.46), and a 5‐item Work/School Satisfaction factor (all CFA loadings > 0.84). The correlations between factors ranged from 0.53 to 0.65 (all large effect size range).

#### 3.1.1 IRT Calibration

Each frequency and support self‐report scale was calibrated separately using the 2‐parameter GRM in IRTPRO 3.1. Overall, the measures with the support needed response option yielded more information than their frequency counterparts.

##### 3.1.1.1 Frequency Scales

For self‐report frequency scales (see Figure 1), we removed 7 items for low information, 7 items for LD, 2 for item thresholds, one item for both LD and age DIF, and one for both LD and model misfit. No items were removed for sex DIF. No items were removed from Work/School Readiness frequency, Work/School Performance frequency, or Mobility frequency based on IRT results.

Of note, the romance factor was entirely dropped. Two items (the ones with language related to engaging in sexual activities) provided the majority of information, and they had poor item thresholds (i.e., whether one answered not at all versus other frequencies yielded the most information with more limited distinction between the other response options). Given our goal to develop continuous multi‐item measures, we considered the scale insufficient to retain overall.

As IRT was our final step in item selection, we also did a careful review of the remaining items after the IRT results and dropped some additional items due to construct validity concerns (primarily resulting from different facets in our conceptual model loading onto the same factor) This included the item “follows a daily schedule” which loaded on Mobility. A couple of items were dropped that were not considered essential given other remaining items and to maintain as much consistency with proxy report as possible. These items performed poorly in proxy report IRT, including an item about traffic signals and one sleep item. Finally, an item on preparing resumes was removed from the Work/School Readiness frequency scale as we believed that the response option would not apply since it is not a regularly repeated activity.

##### 3.1.1.2 Support Scales

For support scales (Figure 2), most items were removed for multiple problems during IRT. Like frequency, no Work/School Readiness or Performance items were removed due to IRT concerns. Overall, more items were flagged for model misfit with support ratings than for frequency. Two items included flags for age‐related DIF and one for sex‐related DIF. Six items included flags for low information. One final item was removed following IRT, focused on following a daily schedule in the due to poor construct validity with the Mobility factor.

##### 3.1.1.3 Satisfaction Scales

**FIGURE 3:**
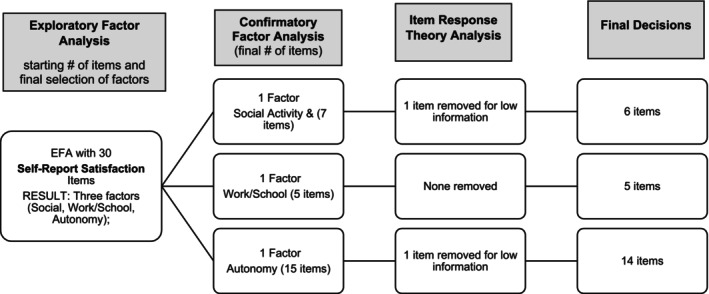
Self‐report satisfaction ratings—Summary of factor analysis and IRT decisions. LD = local dependency.

Because the goal of the Satisfaction scales was to mirror content in the other scales, very few items were removed during IRT (Figure 3). Only two total items were removed, both for low information.

#### 3.1.2 Final Self‐Report REALS Measures and Item Banks

The final 19 self‐report REALS Measures consist of: (1) 8 scales with frequency options covering the domains of: Social Activity, Work Readiness, Work/School Performance, Home Care, Self‐Care, Leisure, Mobility, and Finances, (2) 8 scales with support needed response options in the same domains as the frequency scales, and (3) three satisfaction scales in Social Activity, Autonomy, and Work/School. See Table 4 for a summary of scales and the number of final items per scale. A summary of the discrimination and location parameters for each scale is presented in Table 3. Scoring tables were generated for each scale to transform summed raw scores into their corresponding IRT theta scores and t‐scores. The scoring tables are available upon request for free, together with the REALS Measures themselves.

**TABLE 3:** REALS measures psychometric summary—IRT parameters.

Measure	REALS self‐report					
	Final number of items	Frequency response option		Final number of items	Support response option	
		Slope parameter mean (range)	Location parameter mean (range (*b*_*1*_–*b*_*4*_)		Slope parameter mean (range)	Location parameter mean (range (*b*_*1*_–*b*_*4*_)
Social activity	11	2.04 (1.39–2.47)	−0.18 (−3.39–1.38)	9	2.66 (2.16–3.33)	−0.89 (−2.99–0.45)
Work/School readiness	6	5.34 (2.75–7.80)	0.38 (−0.46–1.12)	7	4.96 (2.79–9.75)	0.25 (−0.81–1.26)
Work/School performance	6	1.76 (1.52–2.07)	−1.31 (−3.32–0.78)	6	2.39 (1.98–2.94)	−1.36 (−3.18–0.25)
Finances	4	2.41 (1.73–3.56)	−0.89 (−2.17–0.52)	4	2.71 (2.32–2.95)	−0.42 (−1.58–0.61)
Home care	4	2.75 (1.40–3.69)	−0.87 (−2.73–0.63)	4	3.17 (1.74–4.28)	−0.84 (−2.37–0.38)
Leisure	4	2.26 (1.00–3.78)	−0.73 (−2.60–1.93)	4	2.90 (1.59–4.17)	−1.15 (−2.48–0.71)
Mobility	3	2.14 (1.61–2.56)	−1.04 (−2.63–0.74)	3	2.67 (2.42–2.99)	−1.05 (−2.54–0.25)
Self‐care	10	1.49 (1.15–1.95)	−1.30 (−3.32–1.20)	10	1.89 (1.45–2.82)	−1.22 (−3.65–0.52)
Satisfaction with social activity	6	3.20 (2.23–3.74)				
Satisfaction with autonomy	14	1.82 (1.40–2.38)				
Satisfaction with work/school	5	3.03 (2.84–3.30)				

Measure	REALS proxy‐report^a^					
	Final number of items	Frequency response option		Final number of items	Support response option	
		Slope parameter mean (range)	Location parameter mean (range (*b*_*1*_–*b*_*4*_)		Slope parameter mean (range)	Location parameter mean (range (*b*_*1*_–*b*_*4*_)
Social activity	9	2.34 (1.34–3.22)	0.23 (−2.04–1.71)	10	2.75 (2.16–3.18)	−0.22 (−2.82–1.05)
Work/School readiness	6	6.49 (1.90–13.39)	0.78 (0.15–1.53)	7	4.83 (2.44–7.71)	1.13 (0.03–1.91)
Work/School performance	6	2.07 (1.55–2.48)	−0.86 (−3.16–0.79)	6	3.02 (2.25–3.41)	−0.41 (−2.36–0.78)
Finances	5	3.16 (2.52–3.76)	−0.17 (−0.96–0.77)	5	3.72 (2.85–4.74)	0.49 (−0.48–1.26)
Home care	4	2.89 (1.38–4.60)	−0.67 (−2.08–0.89)	4	3.09 (1.94–4.53)	−0.17 (−1.83–1.13)
Leisure	3	2.75 (1.76–4.62)	−1.14 (−2.71–0.45)	3	3.45 (2.18–5.63)	−0.91 (−2.15–0.28)
Mobility	4	2.60 (1.74–3.50)	−0.46 (−1.81–1.00)	4	3.39 (2.83–3.84)	−0.02 (−1.37–1.13)
Self‐care				6	3.00 (1.96–4.92)	−0.20 (−2.46–1.1)
Sleep, Diet, and Exercise				4	2.78 (1.03–5.00)	−0.50 (−2.94–1.42)

^a^ Life satisfaction not included in REALS proxy‐report; the slope parameter tells you how well an item discriminates between respondents with low versus high scores. Lower location parameters (or threshold values) indicate that an item is easier to endorse, and higher location parameters indicate that an item is more difficult to endorse.

**TABLE 4:** REALS measures: Ranges of reliability > 0.90 from IRT test information curves.

Measure	Self‐report		Proxy‐report	
	Frequency response	Support response	Frequency response	Support response
	SD	SD	SD	SD
Social activity	−0.5 – +2.0	−3.0 – +1.0	−1.5 – +2.0	−1.5 – +1.5
Work readiness	−1.0 – +1.5	−1.0 – +1.5	−0.5 – +1.5	−0.5 – +2.5
Work/School performance	−3.0 – +1.0	−3.0 – +0.5	−2.5 – +1.0	−2.0 – +1.0
Finances	−2.0 – +0.5	−1.5 – +1.0	−1.0 – +1.0	−0.5 – +1.5
Home care	−2.5 – +1.0	−2.0 – +1.0		
Leisure	−2.5 – +0.5	−2.5 – 0.0	−2.5 – +1.0	−2.5 – +0.5
Mobility	−2.5 – +0.5	−2.5 – +0.5	−1.0 – +0.5	−1.0 – +1.0
Self‐care	−3.0 – +1.0	−3.0 – +0.5	—	−2.0 – +1.5
Sleep, diet, and exercise	—	—	—	−2.0 – +1.0
Satisfaction with social activity	−1.5 – +1.5	—	—	—
Satisfaction with autonomy	−2.0 – +1.5	—	—	—
Satisfaction with work/school	−1.5 – +1.0	—	—	—

**TABLE 5:** Convergent validity inter‐correlations with final REALS self‐report measures.

REALS measure	1	2^a^	3	4	5	6	7	8	9	10	11	12	13	14	15	16	17	18	19
1. Social activity		**0.54**	**0.44**	**0.30**	**0.51**	**0.60**	**0.47**	**0.34**	**0.31**	**0.26**	**0.41**	0.06	**0.30**	**0.32**	**0.31**	**0.21**	**0.24**	**0.51**	−0.00
2. Work readiness	**0.57**			0.18	0.24	**0.49**	0.41*	0.26	0.27	0.05	0.24	0.14	0.02	0.11	0.13	−0.06	0.17	0.21	−0.06
3. Work performance	**0.70**			**0.39**	**0.59**	**0.44**	**0.51**	**0.47**	**0.26**	**0.39**	0.15*	**0.22**	**0.18**	**0.16**	0.07	**0.28**	**0.46**	**0.25**	**0.21**
4. Home care	**0.53**	**0.58**	**0.66**		**0.59**	**0.33**	**0.47**	**0.50**	**0.28**	**0.27**	0.12*	**0.31**	**0.25**	**0.21**	0.08	**0.30**	**0.36**	**0.19**	**0.18**
5. Self‐care	**0.70**	**0.67**	**0.74**	**0.67**		**0.56**	**0.61**	**0.68**	**0.36**	**0.27**	**0.23**	**0.25**	**0.32**	**0.29**	**0.18**	**0.31**	**0.32**	**0.32**	**0.17**
6. Leisure	**0.76**	**0.55**	**0.66**	**0.54**	**0.74**		**0.47**	**0.42**	**0.30**	**0.23**	**0.29**	0.05	**0.28**	**0.33**	**0.22**	**0.28**	**0.20**	**0.37**	0.06
7. Mobility	**0.65**	**0.56**	**0.68**	**0.59**	**0.73**	**0.68**		**0.48**	**0.21**	**0.29**	0.11*	**0.35**	**0.24**	**0.15**	0.06	**0.25**	**0.35**	**0.27**	**0.14**
8. Finances	**0.58**	**0.55**	**0.66**	**0.64**	**0.77**	**0.60**	**0.61**		**0.34**	**0.23**	0.09^+^	**0.29**	**0.27**	**0.24**	0.11*	**0.25**	**0.24**	**0.20**	0.12*
9. Satisfaction with social activity	**0.25**	0.02	**0.22**	**0.34**	**0.34**	**0.26**	0.11*	**0.32**		**0.58**	**0.57**	**0.19**	**0.64**	**0.67**	**0.50**	**0.71**	**0.32**	**0.51**	**0.25**
10. Satisfaction with autonomy	**0.29**	−0.05	**0.38**	**0.36**	**0.31**	**0.30**	**0.26**	**0.26**	**0.58**		**0.47**	**0.22**	**0.49**	**0.47**	**0.35**	**0.54**	**0.42**	**0.39**	**0.23**
11. Satisfaction with work/school	**0.28**	0.10	0.11*	**0.18**	**0.20**	**0.26**	0.06	**0.13**	**0.57**	**0.47**		0.06	**0.44**	**0.52**	**0.66**	**0.48**	**0.22**	**0.48**	**0.19**
**Waisman ADL**																			
12. W‐ADL	**0.45**	**0.49**	**0.52**	**0.54**	**0.61**	**0.46**	**0.62**	**0.68**	**0.19**	**0.22**	0.06		**0.21**	**0.13**	0.05	**0.21**	**0.51**	**0.18**	**0.29**
**WHOQOL**																			
13. Physical	**0.27**	0.03	**0.15**	**0.22**	**0.28**	**0.28**	**0.17**	**0.27**	**0.64**	**0.49**	**0.44**	**0.21**		**0.67**	**0.44**	**0.62**	**0.41**	**0.51**	**0.29**
14. Psychological	**0.22**	−0.00	0.11^+^	**0.18**	**0.24**	**0.27**	0.05	**0.18**	**0.67**	**0.47**	**0.52**	**0.13**	**0.67**		**0.57**	**0.63**	**0.30**	**0.56**	**0.32**
15. Social relationships	**0.16**	−0.04	0.01	**0.12**	**0.14**	**0.17**	0.00	0.07^+^	**0.50**	**0.35**	**0.66**	0.05	**0.44**	**0.57**		**0.49**	**0.16**	**0.44**	**0.20**
16. Environmental	**0.29**	0.00	**0.26**	**0.31**	**0.32**	**0.36**	**0.22**	**0.29**	**0.71**	**0.54**	**0.48**	**0.21**	**0.62**	**0.63**	**0.49**		**0.35**	**0.45**	**0.38**
**SLOF**																			
17. Work skills	**0.51**	**0.65**	**0.63**	**0.55**	**0.53**	**0.47**	**0.54**	**0.51**	**0.32**	**0.42**	**0.22**	**0.51**	**0.41**	**0.30**	**0.16**	**0.35**		**0.39**	**0.33**
18. Interpersonal relationships	**0.47**	0.29^+^	**0.19**	**0.21**	**0.29**	**0.35**	**0.19**	**0.19**	**0.51**	**0.39**	**0.48**	**0.18**	**0.51**	**0.56**	**0.44**	**0.45**	**0.39**		**0.24**
19. Activities	**0.31**	0.29^+^	**0.36**	**0.27**	**0.43**	**0.39**	**0.32**	**0.32**	**0.25**	**0.23**	**0.19**	**0.29**	**0.29**	**0.32**	**0.20**	**0.38**	**0.33**	**0.24**	

*Note*: Associations with frequency and support response options appear in the upper (lower) diagonal; *N* ranges from 401 to 841 depending on the variable. Correlations significant at *p* < 0.001 appear in **boldface**; *p < 0.01; ^+^ *p* < 0.05.

^a^ Range of *N* = 41–72.

**TABLE 6:** Convergent validity inter‐correlations with final REALS proxy report measures.

REALS measure	1	2	3	4	5	6	7	8	9	10	11	12	13	14	15	16	17
1. Social activity		**0.56**	**0.56**	**0.46**		**0.43**	**0.58**	**0.37**		**0.48**	**0.59**	**0.55**	**0.54**	**0.59**	**0.45**	**0.53**	**0.46**
2. Work readiness	**0.66**		**0.62** ^a^	**0.51**		**0.25**	**0.59**	**0.31**		**0.31**	**0.36**	**0.38**	**0.32**	**0.32**	**0.43**	0.18*	**0.31**
3. Work performance	**0.80**	**0.77** ^b^		**0.51**		**0.46**	**0.61**	**0.53**		**0.40**	**0.48**	**0.45**	**0.45**	**0.44**	**0.53**	**0.36**	**0.42**
4. Home care	**0.68**	**0.64**	**0.77**			**0.27**	**0.59**	**0.53**		**0.45**	**0.52**	**0.53**	**0.47**	**0.45**	**0.48**	**0.26**	**0.47**
5. Self‐care	**0.75**	**0.66**	**0.81**	**0.78**													
6. Leisure	**0.67**	**0.46**	**0.67**	**0.55**	**0.64**		**0.39**	**0.34**		**0.28**	**0.29**	**0.26**	**0.29**	**0.28**	**0.27**	**0.29**	**0.26**
7. Mobility	**0.76**	**0.68**	**0.78**	**0.72**	**0.82**	**0.63**		**0.48**		**0.61**	**0.62**	**0.62**	**0.59**	**0.56**	**0.57**	**0.39**	**0.58**
8. Finances	**0.63**	**0.56**	**0.70**	**0.65**	**0.75**	**0.60**	**0.67**			**0.40**	**0.32**	**0.37**	**0.25**	**0.26**	**0.32**	**0.26**	**0.24**
9. Sleep, diet, and exercise	**0.59**	**0.40**	**0.57**	**0.54**	**0.60**	**0.54**	**0.55**	**0.66**									
**Waisman ADL**																	
10. Overall score	**0.71**	**0.64**	**0.74**	**0.69**	**0.82**	**0.64**	**0.79**	**0.80**	**0.56**		**0.81**	**0.86**	**0.78**	**0.68**	**0.75**	**0.43**	**0.25**
**Vineland Adaptive Behavior Scales**																	
11. Adaptive behavior composite	**0.77**	**0.68**	**0.79**	**0.76**	**0.83**	**0.64**	**0.79**	**0.69**	**0.53**	**0.81**		**0.95**	**0.95**	**0.92**	**0.78**	**0.58**	**0.85**
12. Daily living skills	**0.73**	**0.67**	**0.76**	**0.77**	**0.85**	**0.61**	**0.79**	**0.74**	**0.55**	**0.86**	**0.95**		**0.89**	**0.82**	**0.78**	**0.49**	**0.86**
13. Communication	**0.74**	**0.66**	**0.75**	**0.74**	**0.82**	**0.64**	**0.77**	**0.64**	**0.47**	**0.78**	**0.95**	**0.89**		**0.81**	**0.76**	**0.52**	**0.86**
14. Socialization	**0.72**	**0.59**	**0.71**	**0.68**	**0.72**	**0.57**	**0.70**	**0.60**	**0.49**	**0.68**	**0.92**	**0.82**	**0.81**		**0.69**	**0.61**	**0.72**
**SLOF**																	
15. Work skills	**0.69**	**0.65**	**0.79**	**0.71**	**0.75**	**0.56**	**0.71**	**0.66**	**0.49**	**0.75**	**0.78**	**0.78**	**0.76**	**0.69**		**0.54**	**0.23**
16. Interpersonal relationships	**0.59**	**0.37**	**0.47**	**0.42**	**0.46**	**0.41**	**0.46**	**0.37**	**0.39**	**0.43**	**0.58**	**0.49**	**0.52**	**0.61**	**0.54**		0.08^+^
17. Activities	**0.74**	**0.63**	**0.75**	**0.74**	**0.81**	**0.63**	**0.80**	**0.65**	**0.48**	**0.25**	**0.85**	**0.86**	**0.86**	**0.72**	**0.23**	0.08^+^	

*Note*: Associations with frequency and support response options appear in the upper (lower) diagonal; *N* ranges from 307 to 886 depending on the variable. Correlations significant at *p* < 0.001 appear in **boldface**; *p < 0.01; ^+^ *p* < 0.05.

^a^ *N* = 83.

^b^ *N* = 135.

**TABLE 7:** Final REALS measures—Internal reliabilities.

Domain	Self‐report		Proxy report	
	Frequency	Support	Frequency	Support
Relationships				
Social relationships	0.91	0.92	0.91	0.94
Employment				
Work readiness	0.95	0.96	0.93	0.95
Work/School performance	0.80	0.87	0.84	0.92
Autonomy				
Home care	0.83	0.86	0.83	0.86
Self‐care	0.83	0.87		0.90
Leisure	0.76	0.82		
Mobility	0.74	0.80	0.90	0.90
Finances	0.81	0.85	0.90	0.93
Sleep, diet, and exercise				0.89
Satisfaction				
Satisfaction with Social relationships	0.92			
Satisfaction with autonomy	0.91			
Satisfaction with work/school	0.90			

### 3.2 Proxy Report

#### 3.2.1 Factor Analysis

**FIGURE 4:**
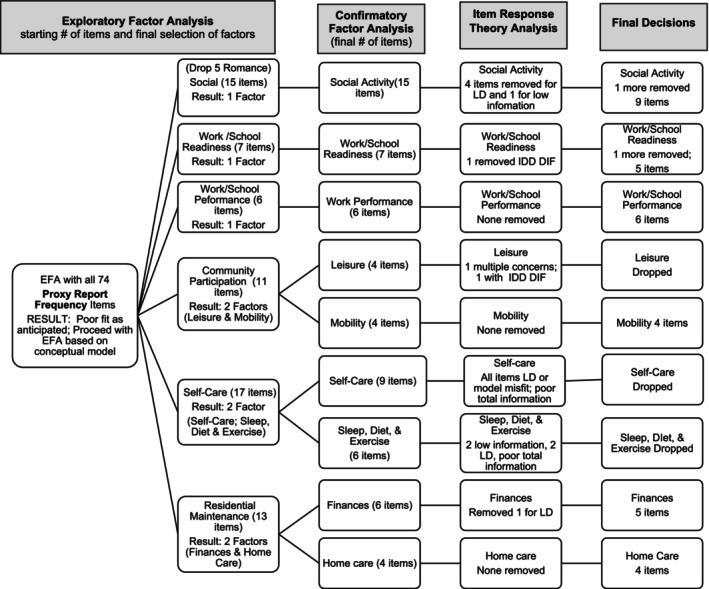
Proxy report frequency ratings—Summary of factor analysis and IRT decisions. LD = local dependency.

**FIGURE 5:**
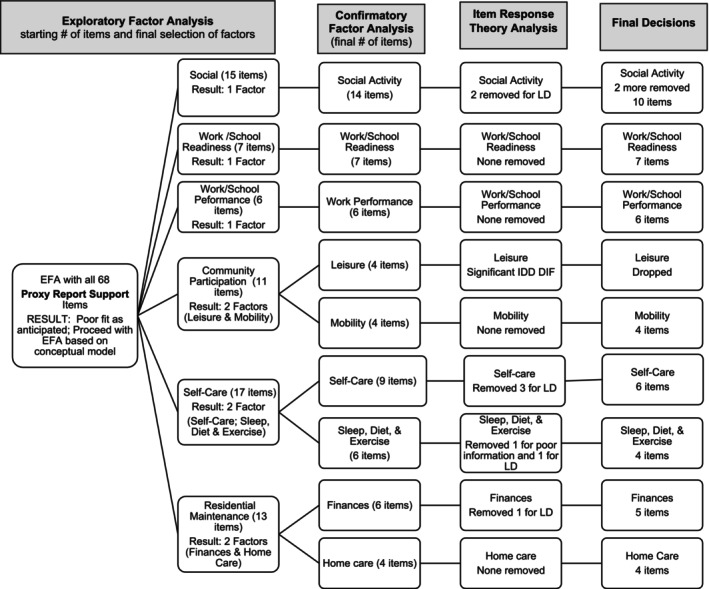
Proxy report support ratings—Summary of factor analysis and IRT decisions. LD = local dependency.

Analysis of proxy reports used the same approach as that for self‐reports (see Tables S10–S15 for factor loadings). Similar to self‐report, EFA with all items together were uninterpretable for both frequency and support scales, so we proceeded with our conceptual model. The conceptual model‐based factor analyses performed well. The proxy report frequency response option (Figure 4) and support response option (Figure 5) scales were almost identical to each other in terms of EFA and CFA results.

Within Social Relationships, we removed Romance items given poor performance in self‐report and a desire for similar construct coverage across self‐ and proxy‐report. The resultant single factor for frequency rating (all CFA factor loadings > 0.53 with the exception of one item loading at 0.43) and support rating (all CFA factor loadings > 0.65) mirrored the Social Activity scale for self‐report. In the employment domain, there was strong support for a single factor for Work/School Readiness frequency items (all CFA loadings > 0.69) as well as for Work/School Readiness support items (all CFA loadings > 0.78). Results were similar for Work/School Performance, with a single factor for frequency (all CFA loadings > 0.74) and a single factor for support (all CFA loadings > 0.82). Also, like self‐report, Community Participation yielded a two‐factor structure with Leisure and Mobility options for both frequency (Leisure CFA all loadings > 0.40; Mobility > 0.73) and support ratings (Leisure CFA all loadings > 0.72; Mobility > 0.85). The Leisure and Mobility factors were correlated 0.50 for frequency ratings and 0.82 for support ratings (both large effect size). The area that differed most from self‐report was Self‐Care. In proxy report, Self‐Care yielded a two‐factor solution for both frequency and support response options, whereas it was one factor for self‐report. For proxy report, items related to sleep, diet, and exercise loaded onto their own factor for both frequency (all CFA loadings > 0.53) and support (all CFA loadings > 0.51 except one item loaded 0.40 that was retained to maintain consistency). Other aspects of self‐care (i.e., hygiene, internet safety, use of medicines and healthcare) loaded onto a separate factor for both frequency (all CFA loadings > 0.57) and support (all CFA loadings > 0.81). The two factors were correlated 0.42 for frequency (medium effect size) and 0.65 for support (large effect size). Residential Maintenance yielded factors for Finances (all CFA loadings > 0.79) and Home Care (all CFA loadings > 0.82) for frequency, and the same factors of Finances (all CFA loadings > 0.71) and Home Care for support (all CFA loadings > 0.88). Finances and Home Care were correlated 0.60 for frequency and 0.73 for support (both large effect size).

#### 3.2.2 IRT Calibration

IRT analyses were completed in the same manner as the self‐report measures. Again, support measures yielded more total information than the frequency measures.

##### 3.2.2.1 Frequency Response Option

No items were removed during IRT for Home Care, Mobility, or Work/School Performance scales. The most common reason to remove items was local dependency with other items (resulting in removal of seven items). No age or sex‐related DIF warranted item removal. However, one item on the Work/School Readiness measure frequency response option related to applying for jobs was removed after DIF analysis revealed it yielded substantially less information in those with IDD (*N* = 440) and compared to those without intellectual disability (*N* = 471). An item on the Leisure scale (does things in daily life that are interesting to them) also provided significantly less information for adults with IDD. An additional Leisure item (attends community events) had a problem with many IRT parameters, leaving just two acceptable items. Therefore, a decision was made to drop the Leisure scale. Both Self‐Care and Sleep, Diet, and Exercise contained items with local dependency, model misfit, or yielded low information, so both subscales were dropped. Finally, an item about disagreements within Social Activity domain was dropped due to concern that it may be less relevant to those who are minimally speaking, and the item on resume preparation within Work/School Readiness was removed like self‐report.

##### 3.2.2.2 Support Response Option

No items were removed during IRT for Work/School Readiness, Work/School Performance, Mobility, and Home Care in support response options. Similar to frequency response options, the primary reason for removing items at this stage was local dependency. The same problems occurred within Leisure for support response options as they did for frequency, again resulting in a decision to remove the scale entirely. The biggest difference between frequency and support proxy scales was in the Self‐Care domain. Whereas these items provided limited total information when rated by frequency, they did provide sufficient information when rated in terms of Support needed and could be retained. Although the content is similar to the Self‐Care scale in self‐report, the proxy version resulted in Self‐Care split into two scales as described above in factor analysis. Finally, two items were removed from Social Activities to maintain consistency in construct coverage across scales (an item on physical space boundaries that did not perform well on other versions) and the item on disagreements; these items were also believed to be too skill‐based upon final review.

#### 3.2.3 Final Proxy‐Report REALS Measures and Item Banks

The final proxy‐report REALS measures consist of 14 brief scales: (1) 6 frequency response options of: Social Activity, Work Readiness, Work/School Performance, Finances, Home Care, and Mobility, and (2) 8 scales with support needed response options in the same domains as frequency as well as Self‐Care (focused on hygiene, medication, and healthcare) and Sleep, Diet, and Exercise. Scoring tables were also generated for the transformation from raw scores to IRT theta scores and *T*‐scores. The primary differences between the proxy report and self‐report scales are the lack of Satisfaction measures (by design) for proxies, no Leisure scale for proxy reporters, two scales rather than one for Self‐Care, and the lack of a frequency option for Self‐Care. The IRT results are summarized in Table 3.

### 3.3 Convergent Validity

Examination of associations between the final REALS Measures and assessments of adaptive behavior using the Vineland Adaptive Behavior Scales, as well as measures of activities of daily living and quality of life were conducted to examine convergent validity. As can be seen in Table 5, self‐report items were significantly associated with activities of daily living, as well as multiple domains of functional outcome. Satisfaction items were particularly associated with better quality of life. Table 6 shows results for proxy reporters, which found medium‐sized and significant correlations with the adaptive behavior composite, daily living skills, socialization, and communication scores on the Vineland Adaptive Behavioral Scales. Significant associations were also observed with measures of functional outcome and activities of daily living. Patterns of association with convergent validity measures were similar for frequency and support response options.

### 3.4 Internal Consistency

All final versions of the REALS Measures had excellent internal consistency (see Table 7). In total, 17 had internal consistency about 0.90, 14 above 0.80. Only two (self‐report frequency Leisure and Mobility) had internal consistencies in the 0.75 range, which is still acceptable.

## 4 Discussion

The REALS Measures are a set of 19 self‐report and 14 proxy‐report questionnaires assessing key aspects of adult life for autistic adults and adults with other IDDs. The Measures were created and refined using a systematic process of item development (MacKenzie et al. 2024) and psychometric analyses with 875 adults with IDDs and 911 caregivers of adults with IDDs. The REALS Measures were developed with expert input from researchers, clinicians, parents/caregivers, and adults with IDDs to focus on the areas of adult life that are both important priorities and potential challenges for adults with IDDs (Bush and Tassé 2017; Esbensen et al. 2010; Gilmore and Cuskelly 2014; Gotham et al. 2015; Hustyi et al. 2015; Taylor and Hodapp 2012). The Measures assess Social Activity, Autonomy, Employment/School, and Life Satisfaction. Each domain can be assessed with a scale that has a frequency (how often an activity is done) or support response options (how much support is needed to complete the activity).

The REALS Measures represent a significant advance in that they were developed specifically for adults, thereby offering coverage of previously neglected areas of adult life. This differs from many of the commonly used measures in adult IDD that were often initially developed for children, or other clinical populations (Berry‐Kravis et al. 2013; Esbensen et al. 2017; Hart et al. 2017; Henninger and Taylor 2013; Koslowski et al. 2016; Nicolaidis et al. 2020). In doing so, it offers scales of employment, which is critical given that employment contributes to many positive outcomes such as higher self‐esteem and financial stability (Faragher, Cass, and Cooper 2005). The REALS Measures also offer continuous measurement to avoid broad categorical descriptions of outcome and promote greater sensitivity to change. Particularly for proxy report, the inclusion of autistic adults with IDDs as well as other IDDs also supports use across service lines that often serve diverse IDD populations. Factor analysis supported separate scales based on the initial conceptual model used to develop REALS items (Social Relationships, Employment/School, Autonomy, and Satisfaction). This allows for more differentiated profiles of adaptation; this may support greater precision in the way we identify and discuss autistic adults needs, which has been called for to reduce ableism that can occur with more global conclusions or summary terms (Bottema‐Beutel et al. 2021). The REALS Measures are also a suite of brief questionnaires (versus a single long measure), which improves efficiency; that is, users can select content areas most relevant to their targeted outcomes and these outcomes can be measured both briefly and precisely.

Further, the majority of REALS Measures are available as either proxy or self‐report. Self‐report encourages self‐determination, whereas proxy report allows the inclusion of those unable to report on themselves and the ability to offer multi‐reporter assessment. To date, most measures of adult outcomes in those with IDD have been proxy‐report (Fujiura 2012; Shogren et al. 2021), but the self‐report REALS Measures can now capture an adult's own viewpoint when possible and enhance self‐determination in goal setting. The REALS Measures also have frequency and support response options for each scale (except for the Satisfaction Measures). This flexibility allows researchers and clinicians to select a response option based on their needs. The frequency option offers traditional assessment of how often someone completes a task. The support option captures the level of support a person required, which may offer a more nuanced understanding of adult life and can be used to measure progress towards more independence. Interestingly, across both proxy and self‐reports and uniformly across content areas, the level of support response sets provided more information (via IRT metrics) than frequency, suggesting that level of support may better capture variability in adult outcomes than the simple frequency. Most items performed well in analyses for frequency and support options, but a few items differ between frequency and support based on better fit. For example, a Social Activity item about having a friend to go to for emotional support is included on the frequency scale but not support. Importantly, both frequency and support scales were correlated with widely used measures of daily living. REALS Satisfaction scales were correlated with existing quality of life measures.

In cognitive interviews for REALS Measure items (see MacKenzie et al. 2024 for more information), proxy reporters voiced that responding to satisfaction items was difficult. Similarly, proxy reporters appeared to have challenges in inferring whether individuals were engaging in leisure activities, which lead to dropping the Leisure scales in proxy report. In general, proxy report items that were based on less observable behaviors or activities were more difficult. Other lessons learned included challenges with dimensional measurement of constructs where the mere presence is so powerful (i.e., sexual activity) and the importance of starting with a strong conceptual model. Finally, the combination of EFA, CFA, and IRT to select items can produce brief, precise, efficient measures with strong internal consistency.

### 4.1 Limitations

The current study does have limitations. First, the sample for this study was mostly White and Non‐Hispanic/Latino, which limits generalizability. It will be important in future research to assess whether the REALS Measures perform similarly across other racial and ethnic groups. Second, among the autism subsample, a majority of self‐report participants were female gender and highly educated, which may not best represent the population of autistic adults. The self‐reporter sample had limited inclusion of adults with other IDD, so more work is needed to understand the application of the REALS measures in IDD by self‐report. Although DIF by IDD status was tested for the proxy report measures, the majority of the IDD sample had co‐occurring autism. Therefore, additional validation work in samples with IDD without autism would be beneficial. Finally, one initial goal was to assess romantic relationships in the social domain, but this aim was not achieved because responses on the romance items were contingent largely on whether the individual was engaging in sexual activity. Future work explicitly focused on romantic and sexual relationships is needed to develop a more dimensional measure of this domain.

Future research with the REALS Measures will include investigating convergent and divergent validity with existing measures of the same domains and assessing change sensitivity in intervention settings. Change sensitivity is particularly key if the REALS Measures are to be used in clinical trials (Esbensen et al. 2017). The REALS Measures are also being compared to Medicaid claims data to assess whether they align with other sources of observational data about adults with IDD.

## Ethics Statement

All procedures performed in studies involving human participants were in accordance with the ethical standards of the University of Pittsburgh Institutional Review Board and with the 1964 Helsinki declaration and its later amendments or comparable ethical standards. No animal subjects were involved in this study.

## Conflicts of Interest

The authors declare no conflicts of interest.

## Supporting information


**Data S1** Supporting Information.

## Data Availability

The data that support the findings of this study are openly available in NIMH Data Archive at https://nda.nih.gov/edit_collection.html?id=3549, reference number 3549.
