# Supplementary material for: The Relationships, Employment, Autonomy, and Life Satisfaction (REALS) Measures for Autistic Adults and Adults With Other Intellectual and Developmental Disabilities: Psychometric Testing of the Self‐Report and Proxy Versions
Source: Autism Res. 2025 Feb 12;18(3):583–603. doi: 10.1002/aur.70002 (PMC11928910; doi:10.1002/aur.70002)
Supplement: Supplementary file 1 — Data S1 Supporting Information. [file AUR-18-583-s001.docx]

**Supplemental tables**

**Supplemental Table 1. REALS caregiver-report scores by subgroups**

| **REALS scale** | **Gender** |  |  |
| --- | --- | --- | --- |
|  | **Male** | **Female** |  |
|  | **M (SD)** | **M (SD)** |  |
| Social-Frequency | -0.16 (0.96) | 0.20 (0.87) |  |
| Social- Support | -0.16 (0.94) | 0.24 (0.93) |  |
| Leisure-Frequency | 0.01 (0.89) | -0.05 (0.82) |  |
| Leisure-Support | -0.06 (0.91) | 0.10 (0.84) |  |
| Mobility-Frequency | -0.10 (0.94) | 0.14 (0.86) |  |
| Mobility-Support | -0.14 (0.93) | 0.20 (0.91) |  |
| Self-care- Support | -0.19 (0.90) | 0.26 (0.97) |  |
| Sleep Diet Exercise-Support | -0.04 (0.85) | 0.08 (0.91) |  |
| Home Care-Frequency | -0.06 (0.91) | 0.02 (0.87) |  |
| Home Care-Support | -0.13 (0.89) | 0.21 (0.95) |  |
| Finances-Frequency | -0.19 (0.99) | 0.05 (0.86) |  |
| Finances-Support | -0.15 (0.89) | 0.22 (0.94) |  |
| Work/School Performance-Frequency | -0.05 (0.91) | 0.03 (0.92) |  |
| Work/School Performance-Support | -0.13 (0.97) | 0.19 (0.90) |  |
| Work Readiness-Frequency | -0.21 (0.79) | -0.01 (0.92) |  |
| Work Readiness-Support | -0.13 (0.82) | 0.21 (0.10) |  |
|  |  |  |  |
| **REALS Scale** | **Autism only group** | **IDD group** |  |
|  | **M (SD)** | **M (SD)** |  |
| Social-Frequency | 0.13 (0.89) | -0.19 (0.96) |  |
| Social- Support | 0.34 (0.94) | -0.37 (0.83) |  |
| Leisure-Frequency | 0.09 (0.85) | -0.11 (0.88) |  |
| Leisure-Support | 0.30 (0.82) | -0.32 (0.84) |  |
| Mobility-Frequency | 0.26 (0.86) | -0.33 (0.88) |  |
| Mobility-Support | 0.43 (0.91) | -0.47 (0.72) |  |
| Self-care- Support | 0.48 (0.89) | -0.52 (0.71) |  |
| Sleep Diet Exercise-Support | 0.20 (0.96) | -0.22 (0.75) |  |
| Home Care-Frequency | -0.01 (0.86) | -0.03 (0.94) |  |
| Home Care-Support | 0.31 (0.90) | -0.32 (0.84) |  |
| Finances-Frequency | 0.14 (0.84) | -0.37 (0.99) |  |
| Finances-Support | 0.45 (0.92) | -0.48 (0.67) |  |
| Work/School Performance-Frequency | 0.19 (0.83) | -0.27 (0.95) |  |
| Work/School Performance-Support | 0.38 (0.89) | -0.49 (0.81) |  |
| Work Readiness-Frequency | 0.001 (0.85) | -0.24 (0.83) |  |
| Work Readiness-Support | 0.42 (0.99) | -0.31 (0.70) |  |
|  |  |  |  |
| **REALS scale** | **Not employed or in school** | **Employed or in school** |  |
|  | **M (SD)** | **M (SD)** |  |
| Social-Frequency | -0.26 (0.91) | 0.27 (0.90) |  |
| Social- Support | -0.34 (0.87) | 0.45 (0.88) |  |
| Leisure-Frequency | -0.14 (0.87) | 0.18 (0.83) |  |
| Leisure-Support | -0.24 (0.88) | 0.32 (0.78) |  |
| Mobility-Frequency | -0.34 (0.90) | 0.38 (0.78) |  |
| Mobility-Support | -0.36 (0.82) | 0.49 (0.86) |  |
| Self-care- Support | -0.34 (0.87) | 0.45 (0.85) |  |
| Sleep Diet Exercise-Support | -0.23 (0.81) | 0.31 (0.90) |  |
| Home Care-Frequency | -0.18 (0.95) | 0.18 (0.80) |  |
| Home Care-Support | -0.32 (0.87) | 0.45 (0.81) |  |
| Finances-Frequency | -0.35 (0.96) | 0.21 (0.83) |  |
| Finances-Support | -0.34 (0.79) | 0.45 (0.90) |  |
| Work/School Performance-Frequency | -0.72 (1.00) | 0.18 (0.79) |  |
| Work/School Performance-Support | -0.75 (0.90) | 0.28 (0.81) |  |
| Work Readiness-Frequency | -0.18 (0.84) | 0.30 (0.82) |  |
| Work Readiness-Support | -0.04 (0.91) | 0.38 (0.76) |  |
|  |  |  |  |
| **REALS scale** | **Age 18-25** | **Age 26-40** | **Age 41-84** |
|  | **M (SD)** | **M (SD)** | **M (SD)** |
| Social-Frequency | -0.11 (0.10) | -0.01 (0.92) | 0.04 (0.90) |
| Social- Support | -0.19 (0.88) | 0.07 (1.01) | 0.08 (0.93) |
| Leisure-Frequency | 0.02 (0.88) | -0.05 (0.90) | -0.004 (0.83) |
| Leisure-Support | -0.06 (0.88) | -0.004 (0.93) | 0.04 (0.86) |
| Mobility-Frequency | -0.18 (0.85) | 0.14 (0.94) | -0.01 (0.93) |
| Mobility-Support | -0.23 (0.83) | 0.20 (0.99) | 0.01 (0.93) |
| Self-care- Support | -0.24 (0.83) | 0.19 (1.03) | 0.01 (0.93) |
| Sleep Diet Exercise-Support | -0.17 (0.80) | 0.04 (0.99) | 0.09 (0.85) |
| Home Care-Frequency | -0.20 (0.91) | 0.06 (0.87) | 0.07 (0.89) |
| Home Care-Support | -0.19 (0.84) | 0.09 (0.98) | 0.09 (0.92) |
| Finances-Frequency | -0.34 (0.90) | 0.05 (0.97) | -0.04 (0.92) |
| Finances-Support | -0.16 (0.80) | 0.16 (1.0)2 | -0.03 (0.94) |
| Work/School Performance-Frequency | -0.28 (0.97) | 0.22 (0.82) | 0.03 (0.87) |
| Work/School Performance-Support | -0.36 (0.92) | 0.38 (0.92) | 0.02 (0.89) |
| Work Readiness-Frequency | -0.08 (0.80) | -0.20 (0.92) | -0.16 (0.84) |
| Work Readiness-Support | 0.02 (0.80) | 0.01 (1.04) | -0.05 (0.90) |

Supplemental Table 2. REALS self-report scores by subgroups

| **REALS score** | **Gender** |  |  |
| --- | --- | --- | --- |
|  | **Male** | **Female** |  |
|  | **M (SD)** | **M (SD)** |  |
| Social-Frequency | -0.01 (0.93) | 0.05 (0.99) |  |
| Social- Support | -0.08 (0.94) | 0.08 (0.95) |  |
| Leisure-Frequency | -0.02 (0.84) | 0.05 (0.89) |  |
| Leisure-Support | -0.06 (0.84) | 0.08 (0.87) |  |
| Mobility-Frequency | -0.05 (0.84) | 0.08 (0.86) |  |
| Mobility-Support | -0.09 (0.88) | 0.10 (0.86) |  |
| Self-care- Frequency | -0.05 (0.93) | 0.11 (0.91) |  |
| Self-care- Support | -0.09 (0.93) | 0.14 (0.92) |  |
| Home Care-Frequency | -0.03 (0.92) | 0.08 (0.89) |  |
| Home Care-Support | -0.06 (0.93) | 0.09 (0.88) |  |
| Finances-Frequency | -0.03 (0.94) | 0.09 (0.85) |  |
| Finances-Support | -0.08 (0.96) | 0.12 (0.87) |  |
| Work/School Performance-Frequency | -0.05 (0.93) | 0.13 (0.86) |  |
| Work/School Performance-Support | -0.12 (0.95) | 0.13 (0.87) |  |
| Work Readiness-Frequency | 0.31 (0.80) | 0.21 (0.98) |  |
| Work Readiness-Support | 0.14 (0.93) | 0.12 (0.98) |  |
| Autonomy Satisfaction | 0.15 (0.93) | 0.02 (0.93) |  |
| Social Satisfaction | 0.06 (0.92) | 0.02 (0.98) |  |
| Work/School Satisfaction | 0.02 (0.91) | 0.06 (0.91) |  |
|  |  |  |  |
| **REALS score** | **Autism only group** | **IDD group** |  |
|  | **M (SD)** | **M (SD)** |  |
| Social-Frequency | -0.07 (0.90) | 0.34 (1.01) |  |
| Social- Support | 0.09 (0.92) | -0.44 (0.91) |  |
| Leisure-Frequency | -0.02 (0.83) | 0.12 (0.96) |  |
| Leisure-Support | 0.09 (0.98) | -0.45 (0.91) |  |
| Mobility-Frequency | 0.05 (0.84) | -0.21 (0.85) |  |
| Mobility-Support | 0.15 (0.81) | -0.64 (0.82) |  |
| Self-care- Frequency | 0.03 (0.87) | -0.04 (1.05) |  |
| Self-care- Support | 0.12 (0.87) | -0.55 (0.98) |  |
| Home Care-Frequency | -0.01 (0.90) | 0.06 (0.88) |  |
| Home Care-Support | 0.10 (0.88) | -0.46 (0.85) |  |
| Finances-Frequency | 0.07 (0.86) | -0.26 (0.95) |  |
| Finances-Support | 0.11 (0.90) | -0.53 (0.78) |  |
| Work/School Performance-Frequency | 0.05 (0.87) | -0.01 (0.90) |  |
| Work/School Performance-Support | 0.13 (0.86) | -0.63 (0.88) |  |
| Work Readiness-Frequency | 0.06 (0.87) | 0.39 (0.85) |  |
| Work Readiness-Support | 0.30 (1.07) | -0.01 (0.74) |  |
| Autonomy Satisfaction | -0.09 (0.89) | 0.37 (1.09) |  |
| Social Satisfaction | -0 .04 (0.93) | 0.21 (1.00) |  |
| Work/School Satisfaction | 0.002 (0.92) | 0.1 (0.94) |  |
|  |  |  |  |
| **REALS score** | **Not employed or in school** | **Employed or in school** |  |
|  | **M (SD)** | **M (SD)** |  |
| Social-Frequency | -0.18 (0.95) | 0.12 (0.91) |  |
| Social- Support | -0.32 (0.95) | 0.19 (0.89) |  |
| Leisure-Frequency | -0.06 (0.85) | 0.04 (0.86) |  |
| Leisure-Support | -0.24 (0.88) | 0.13 (0.83) |  |
| Mobility-Frequency | -0.26 (0.88) | 0.17 (0.78) |  |
| Mobility-Support | -0.38 (0.85) | 0.24 (0.79) |  |
| Self-care- Frequency | -0.18 (0.96) | 0.13 (0.85) |  |
| Self-care- Support | -0.31 (0.89) | 0.18 (0.90) |  |
| Home Care-Frequency | -0.12 (0.93) | 0.08 (0.87) |  |
| Home Care-Support | -0.30 (0.89) | 0.18 (0.86) |  |
| Finances-Frequency | -0.23 (0.99) | 0.13 (0.80) |  |
| Finances-Support | -0.43 (0.85) | 0.25 (0.85) |  |
| Work/School Performance-Frequency | -0.62 (1.12) | 0.11 (0.82) |  |
| Work/School Performance-Support | -0.67 (0.94) | 0.08 (0.87) |  |
| Work Readiness-Frequency | 0.13 (0.89) | 0.65 (0.60) |  |
| Work Readiness-Support | 0.18 (1.01) | 0.24 (0.68) |  |
| Autonomy Satisfaction | -0.17 (1.01) | 0.10 (0.89) |  |
| Social Satisfaction | -0.13 (1.02) | 0.08 (0.89) |  |
| Work/School Satisfaction | -0.45 (1.01) | 0.28 (0.75) |  |
|  |  |  |  |
| **REALS score** | **Age 18-27** | **Age 28-40** | **Age 41-78** |
| Social-Frequency | 0.16 (0.87) | 0.11 (0.90) | -0.23 (0.99) |
| Social- Support | -0.11 (0.91) | 0.02 (0.91) | 0.06 (1.01) |
| Leisure-Frequency | 0.15 (0.82) | 0.07 (0.85) | -0.21 (0.86) |
| Leisure-Support | -0.12 (0.85) | -0.02 (0.86) | 0.10 (0.89) |
| Mobility-Frequency | -0.13 (0.83) | 0.08 (0.80) | 0.06 (0.88) |
| Mobility-Support | -0.28 (0.90) | 0.01 (0.80) | 0.27 (0.82) |
| Self-care- Frequency | -0.01 (0.86) | 0.04 (0.95) | 0.01 (0.91) |
| Self-care- Support | -0.23 (0.88) | -0.01 (0.93) | 0.21 (0.92) |
| Home Care-Frequency | -0.001 (0.89) | 0.01 (0.90) | 0.01 (0.91) |
| Home Care-Support | -0.18 (0.87) | -0.05 (0.88) | 0.21 (0.91) |
| Finances-Frequency | -0.13 (0.92) | 0.09 (0.82) | 0.04 (0.90) |
| Finances-Support | -0.25 (0.85) | -0.04 (0.88) | 0.25 (0.93) |
| Work/School Performance-Frequency | -0.10 (0.90) | 0.09 (0.82) | 0.12 (0.90) |
| Work/School Performance-Support | -0.30 (0.88) | 0.04 (0.91) | 0.23 (0.86) |
| Work Readiness-Frequency | 0.26 (0.46) | 0.49 (1.34) | -0.27 (1.13) |
| Work Readiness-Support | 0.22 (0.87) | -0.08 (1.17) | 0.43 (1.05) |
| Autonomy Satisfaction | 0.22 (0.92) | -0.03 (0.94) | -0.20 (0.93) |
| Social Satisfaction | 0.20 (0.95) | 0.03 (0.89) | -0.20 (0.96) |
| Work/School Satisfaction | 0.01 (0.90) | 0.03 (0.91) | -0.03 (0.96) |

**Table S3. REALS Social Relationships Scales, Self-report Factor Analysis**

| **Social Relationships Frequency** | | | | |  | **Social Relationships Support** | | |
| --- | --- | --- | --- | --- | --- | --- | --- | --- |
|  | EFA | | CFA | |  |  | EFA | CFA |
|  | 2-FACTOR | | 2-FACTOR | |  |  | 1-FACTOR | 1-FACTOR |
|  | **F1** | **F2** | **F1** | **F2** |  |  | **F1** | **F1** |
| F_013 | 0.679* | -0.212* | 0.691* |  |  | S_013 | 0.809* | 0.84* |
| F_014 | 0.672* | -0.155* | 0.68* |  |  | S_014 | 0.735* | 0.822* |
| F_016 | 0.688* | -0.061 | 0.606* |  |  | S_016 | 0.740* | 0.777* |
| F_017 | 0.703* | 0.029 | 0.739* |  |  | S_017 | 0.689* | 0.756* |
| F_018 | 0.723* | -0.07 | 0.737* |  |  | S_018 | 0.786* | 0.803* |
| F_021 | 0.839* | -0.09 | 0.777* |  |  | S_021 | 0.823* | 0.83* |
| F_023 | 0.761* | 0.026 | 0.768* |  |  | S_023 | 0.822* | 0.848* |
| F_024 | 0.786* | 0.03 | 0.754* |  |  | S_024 | 0.787* | 0.836* |
| F_025 | 0.576* | 0.12 | 0.675* |  |  | S_025 | 0.680* | 0.713* |
| F_036 | 0.777* | -0.042 | 0.796* |  |  | S_036 | 0.812* | 0.817* |
| F_037 | 0.769* | 0.065 | 0.746* |  |  | S_037 | 0.760* | 0.787* |
| F_038 | 0.643* | 0.083 | 0.641* |  |  |  |  |  |
| F_039 | 0.735* | 0.071 | 0.766* |  |  | S_039 | 0.720* | 0.828* |
| F_040 | 0.608* | -0.143* | 0.546* |  |  | S_040 | 0.666* | 0.748* |
| F_041 | 0.794* | 0.062 | 0.766* |  |  | S_041 | 0.760* | 0.768* |
| F_042 | 0.361* | -0.138* |  |  |  | S_042 | 0.590* | 0.671* |
| F_044 | 0.174* | 0.628* |  | 0.824* |  |  |  |  |
| F_045 | 0.234* | 0.515* |  | 0.709* |  |  |  |  |
| F_046 | -0.017 | 0.962* |  | 0.883* |  |  |  |  |
| F_047 | 0.018 | 0.896* |  | 0.951* |  |  |  |  |
|  | CORR | 0.401 | CORR | 0.502 |  |  |  |  |

**Table S4. REALS Community Participation scales, self-report factor analysis**

| **Community Participation Frequency** | | | | |  | **Community Participation Support** | | | | |
| --- | --- | --- | --- | --- | --- | --- | --- | --- | --- | --- |
|  | EFA  2-FACTOR | | CFA  2-FACTOR | |  |  | EFA  2-FACTOR | | CFA  2-FACTOR | |
|  | F1 | F2 | F1 | F2 |  |  | F1 | F2 | F1 | F2 |
| F_032 | 0.781* | 0 | 0.716* |  |  | S_032 | 0.830* | 0.016 | 0.827* |  |
| F_033 | 0.676* | 0.044 | 0.675* |  |  | S_033 | 0.893* | -0.088 | 0.822* |  |
| F_034 | 0.629* | -0.062 | 0.651* |  |  | S_034 | 0.934* | -0.202* | 0.808* |  |
| F_074 | 0.845* | -0.006 | 0.76* |  |  | S_074 | 0.814* | 0 | 0.85* |  |
| F_097 | 0.750* | 0.014 | 0.687* |  |  | S_097 | 0.674* | 0.08 | 0.862* |  |
| F_098 | 0.336* | 0.511* |  | 0.732* |  | S_098 | 0.489* | 0.460* |  | 0.849* |
| F_099 | 0.469* | 0.168* | 0.561* |  |  | S_099 | 0.630* | 0.078 |  | 0.697* |
| F_107 | -0.146 | 0.924* |  | 0.756* |  | S_107 | -0.006 | 0.923* |  | 0.776* |
| F_108 | 0.004 | 0.867* |  | 0.86* |  | S_108 | 0.331* | 0.630* |  | 0.887* |
| F_110 | 0.471* | -0.04 | 0.488* |  |  | S_110 | 0.661* | 0.04 |  |  |
| F_113 | 0.056 | 0.717* |  | 0.764* |  | S_113 | 0.216* | 0.647* |  | 0.818* |
|  | CORR | 0.465 | CORR | 0.565 |  |  | CORR | 0.583 | CORR | 0.849 |

Table S5. REALS Self-Care, self-report factor analysis

| **Frequency** | |  |  | **Support** | |  |
| --- | --- | --- | --- | --- | --- | --- |
| **Self-Care Frequency** | | |  | **Self-Care Support** | | |
|  | EFA | CFA |  |  | EFA | CFA |
|  | 1-FACTOR | 1- FACTOR |  |  | 1-FACTOR | 1-FACTOR |
| F_062 | 0.602* | 0.607* |  | S_062 | 0.720* | 0.759* |
| F_063 | 0.612* | 0.622* |  | S_063 | 0.633* | 0.68* |
| F_064 | 0.564* | 0.612* |  | S_064 | 0.572* | 0.743* |
| F_069 | 0.575* | 0.537* |  | S_069 | 0.607* | 0.63* |
| F_070 | 0.621* | 0.618* |  | S_070 | 0.659* | 0.616* |
| F_071 | 0.611* | 0.642* |  | S_071 | 0.721* | 0.86* |
| F_072 | 0.650* | 0.603* |  | S_072 | 0.668* | 0.679* |
| F_073 | 0.607* | 0.647* |  | S_073 | 0.729* | 0.841* |
| F_90A | 0.771* | 0.724* |  | S_90A | 0.760* | 0.773* |
| F_90B | 0.775* | 0.743* |  | S_90B | 0.859* | 0.843* |
| F_091 | 0.807* | 0.817* |  | S_091 | 0.823* | 0.835* |
| F_092 | 0.817* | 0.839* |  | S_092 | 0.844* | 0.871* |
| F_093 | 0.682* | 0.714* |  | S_093 | 0.822* | 0.816* |
| F_094 | 0.657* | 0.75* |  | S_094 | 0.761* | 0.823* |
| F_095 | 0.606* | 0.567* |  | S_095 | 0.669* | 0.714* |
| F_096 | 0.652* | 0.672* |  | S_096 | 0.742* | 0.741* |
| F_104 | 0.524* | 0.554* |  |  |  |  |

**Table S6. REALS Residential Maintenance scales, self-report factor analysis**

| **Residential Maintenance Frequency** | | | | |  | **Residential Maintenance Support** | | | | |
| --- | --- | --- | --- | --- | --- | --- | --- | --- | --- | --- |
|  | EFA | | CFA | |  |  | EFA | | CFA | |
|  | F1 | F2 | F1 | F2 |  |  | F1 | F2 | F1 | 2F |
| F_060 | -0.101 | 0.896* |  | 0.832 |  | S_060 | -0.011 | 0.881* |  | 0.848* |
| F_076 | 0.897* | -0.002 | 0.845* |  |  | S_076 | 0.928* | -0.056 | 0.883* |  |
| F_077 | 0.794* | 0.03 | 0.818* |  |  | S_077 | 0.823* | 0.001 | 0.802* |  |
| F_078 | 0.919* | -0.031 | 0.828* |  |  | S_078 | 0.911* | 0.003 | 0.901* |  |
| F_079 | 0.636* | 0.253* | 0.813* |  |  | S_079 | 0.606* | 0.340* | 0.843* |  |
| F_080 | 0.550* | 0.263* | 0.747* |  |  | S_080 | 0.625* | 0.253* | 0.808* |  |
| F_083 | 0.026 | 0.735* |  | 0.811* |  | S_083 | 0.380* | 0.537* |  | 0.88* |
| F_084 | 0.061 | 0.786* |  | 0.787* |  | S_084 | 0.388* | 0.571* |  | 0.89* |
| F_085 | 0.075 | 0.694* |  | 0.775* |  | S_085 | 0.267* | 0.611* |  | 0.786* |
| F_086 | -0.001 | 0.802* |  | 0.82* |  | S_086 | -0.09 | 0.865* |  | 0.807* |
| F_087 | -0.029 | 0.788* |  | 0.825* |  | S_087 | 0.134* | 0.782* |  | 0.837* |
| F_101 | 0.255* | 0.360* |  |  |  | S_101 | 0.362* | 0.377* |  |  |
| F_103 | 0.177* | 0.221* |  |  |  | S_103 | 0.253* | 0.450* |  | 0.672* |
|  | CORR | 0.614i | CORR | 0.613 |  |  | CORR | 0.559 | CORR | 0.76 |

**Table S7. REALS Work/School Performance scales, self-report factor analysis**

| **Work/School Performance** | | |  | **Work/School Performance** | | |
| --- | --- | --- | --- | --- | --- | --- |
| **Frequency** | | |  | **Support** | | |
|  | EFA | CFA |  |  | EFA | CFA |
|  | F1 | F1 |  |  | F1 | F1 |
| F_050 | 0.700* | 0.676* |  | S_050 | 0.834* | 0.823* |
| F_052 | 0.665* | 0.625* |  | S_052 | 0.786* | 0.733* |
| F_053 | 0.755* | 0.662* |  | S_053 | 0.775* | 0.81* |
| F_054 | 0.684* | 0.782* |  | S_054 | 0.814* | 0.805* |
| F_055 | 0.675* | 0.652* |  | S_055 | 0.720* | 0.786* |
| F_056 | 0.781* | 0.765* |  | S_056 | 0.869* | 0.835* |

**Table S8. REALS Work Readiness scales, self-report factor analysis**

| **Work/School Readiness** | | |  | **Work/School Readiness** | | |
| --- | --- | --- | --- | --- | --- | --- |
| **Frequency** |  |  |  | **Support** |  |  |
|  | EFA | CFA |  |  | EFA | CFA |
|  | F1 | F1 |  |  | F1 | F1 |
| F_027 | 0.937* | 0.961 |  | F_027 | 0.937* | 0.961* |
| F_028 | 0.970* | 0.959 |  | F_028 | 0.970* | 0.959* |
| F_029 | 0.871* | 0.922 |  | F_029 | 0.871* | 0.922* |
| F_030 | 0.751* | 0.847 |  | F_030 | 0.751* | 0.847* |
| F_048 | 0.932* | 0.939 |  | F_048 | 0.932* | 0.939* |
| F_100 | 0.891* | 0.922 |  | F_100 | 0.891* | 0.922* |
| F_147 | 0.860* | 0.908 |  |  |  |  |

**S9. REALS Satisfaction scales self-report factor analysis**

| EFA | | | |  | CFA | | |
| --- | --- | --- | --- | --- | --- | --- | --- |
|  | F1 | F2 | F3 |  | F1 | F2 | F3 |
| SAT114 | 0.226 | 0.608* | 0.097 |  |  | 0.913* |  |
| SAT115 | 0.226 | 0.625* | 0.034 |  |  | 0.889* |  |
| SAT116 | 0.064 | 0.817* | 0.062 |  |  | 0.84* |  |
| SAT117 | -0.061 | 0.899* | -0.015 |  |  | 0.913* |  |
| SAT118 | 0.002 | 0.886* | 0.003 |  |  | 0.872* |  |
| SAT123a | 0.547* | 0.061 | 0.131 |  | 0.637* |  |  |
| SAT123b | 0.452* | 0.15 | -0.002 |  | 0.461* |  |  |
| SAT124 | 0.781* | 0.004 | -0.086 |  | 0.808* |  |  |
| SAT125 | 0.751* | 0.063 | -0.071 |  | 0.844* |  |  |
| SAT126 | 0.546* | -0.1 | 0.294 |  | 0.632* |  |  |
| SAT127 | 0.754* | 0.012 | 0.006 |  | 0.776* |  |  |
| SAT128 | 0.567* | -0.115 | 0.263 |  | 0.736* |  |  |
| SAT129 | 0.479* | 0.029 | 0.164 |  | 0.767* |  |  |
| SAT130 | 0.69* | -0.127 | 0.207 |  | 0.606* |  |  |
| SAT131 | 0.582* | 0.235 | -0.076 |  | 0.774* |  |  |
| SAT132 | 0.464* | 0.096 | 0.198 |  | 0.619* |  |  |
| SAT134 | 0.528* | -0.051 | 0.308* |  | 0.681* |  |  |
| SAT135 | 0.761* | 0.014 | 0.006 |  | 0.796* |  |  |
| SAT136 | 0.614* | 0.136 | -0.055 |  | 0.649* |  |  |
| SAT137 | 0.376* | 0.564* | -0.091 |  |  | 0.86* |  |
| SAT138 | 0.586* | 0.231 | 0.09 |  | 0.833* |  |  |
| SAT139 | 0.466* | 0.046 | 0.221 |  |  |  |  |
| SAT140 | 0.38* | 0.178 | 0.255 |  |  |  |  |
| SAT141 | 0.303 | -0.016 | 0.455* |  |  |  |  |
| SAT142 | 0.067 | 0.061 | 0.777* |  |  |  | 0.857* |
| SAT143 | -0.035 | 0.113 | 0.85* |  |  |  | 0.877* |
| SAT144 | 0.08 | 0.077 | 0.734* |  |  |  | 0.879* |
| SAT145 | 0.152 | 0.008 | 0.735* |  |  |  | 0.856* |
| SAT146 | -0.006 | -0.001 | 0.831* |  |  |  | 0.849* |
| SAT148 | -0.004 | 0.883* | 0.013 |  |  | 0.869* |  |
|  | CORR | f1 | f2 |  | CORR | f2 | F3 |
|  | f2 | 0.54 | 1 |  | f1 | 0.613 | 0.651 |
|  | f3 | 0.498 | 0.43 |  | f2 |  | 0.537 |

**Table S10. REALS Social Relationship Scale, proxy report factor analysis**

| **Proxy report Social frequency** | | |  | **Proxy report Social support** | | |
| --- | --- | --- | --- | --- | --- | --- |
|  | EFA | CFA |  |  | EFA | CFA |
|  | F1 | F1 |  |  | F1 | F1 |
| F_013 | 0.511* | 0.712* |  | S_013 | 0.777* | 0.876* |
| F_014 | 0.405* | 0.692* |  | S_014 | 0.689* | 0.874* |
| F_016 | 0.706* | 0.731* |  | S_016 | 0.793* | 0.873* |
| F_017 | 0.676* | 0.732* |  | S_017 | 0.76* | 0.841* |
| F_018 | 0.673* | 0.655* |  | S_018 | 0.734* | 0.764* |
| F_021 | 0.719* | 0.76* |  | S_021 | 0.813* | 0.849* |
| F_023 | 0.752* | 0.743* |  | S_023 | 0.8* | 0.829* |
| F_024 | 0.803* | 0.863* |  | S_024 | 0.817* | 0.863* |
| F_025 |  |  |  | S_025 |  |  |
| F_036 | 0.802* | 0.792* |  | S_036 | 0.817* | 0.873* |
| F_037 | 0.754* | 0.807* |  | S_037 | 0.809* | 0.874* |
| F_038 | 0.728* | 0.775* |  |  |  |  |
| F_039 | 0.836* | 0.852* |  | S_039 | 0.808* | 0.804* |
| F_040 | 0.548* | 0.538* |  | S_040 | 0.673* | 0.651* |
| F_041 | 0.846* | 0.829* |  | S_041 | 0.823* | 0.84* |
| F_042 | 0.337* | 0.43* |  | S_042 | 0.563* | 0.657* |
| F_044 |  |  |  |  |  |  |
| F_045 |  |  |  |  |  |  |
| F_046 |  |  |  |  |  |  |
| F_047 |  |  |  |  |  |  |

**Table S11. REALS Community Participation scales, proxy report factor analysis**

| **Community Participation Frequency** | | | | |  | **Community Participation Support** | | | | |
| --- | --- | --- | --- | --- | --- | --- | --- | --- | --- | --- |
|  | EFA | | CFA | |  |  | EFA | | CFA | |
|  | F1 | F2 | F1 | F2 |  |  | F1 | F2 | F1 | F2 |
| F_032 | 0.75* |  | 0.754* |  |  | S_032 | 0.854* |  | 0.827* |  |
| F_033 | 0.298* |  | 0.509* |  |  | S_033 | 0.302* |  | 0.757* |  |
| F_034 |  |  |  |  |  | S_034 |  |  |  |  |
| F_074 | 0.979* |  | 0.814* |  |  | S_074 | 0.916* |  | 0.834* |  |
| F_097 | 0.702* |  | 0.69* |  |  | S_097 | 0.796* |  | 0.729* |  |
| F_098 |  | 0.671* |  | 0.732* |  | S_098 |  | 0.759* |  | 0.857* |
| F_099 |  |  |  |  |  | S_099 |  |  |  |  |
| F_107 |  | 0.891* |  | 0.884* |  | S_107 |  | 0.913* |  | 0.864* |
| F_108 |  | 0.697* |  | 0.854* |  | S_108 |  | 0.795* |  | 0.908* |
| F_110 |  |  |  |  |  | S_110 |  |  |  |  |
| F_113 |  | 0.847* |  | 0.822* |  | S_113 |  | 0.91* |  | 0.886* |
|  | CORR | 0.412 | CORR | 0.505 |  |  | CORR | 0.702 | CORR | 0.825 |

Ta**ble S12. REALS Selfcare scales, proxy report factor analysis**

|  | **Proxy Self-Care Frequency** | | | |  |  | **Proxy Self-Care Support** | | | |
| --- | --- | --- | --- | --- | --- | --- | --- | --- | --- | --- |
|  | EFA | | CFA | |  |  | EFA | | CFA | |
|  | F1 | F2 | F1 | F2 |  |  | F1 | F2 | F1 | F2 |
| F_062 | 0.663* | -0.113 | 0.76* |  |  | S_062 | 0.002 | 0.789* | 0.84* |  |
| F_063 | 0.723* | -0.187* | 0.767* |  |  | S_063 | 0.127* | 0.731* | 0.858* |  |
| F_064 | 0.461* | 0.08 |  |  |  | S_064 | 0.455* | 0.265* |  |  |
| F_069 | 0.173* | 0.375* |  | 0.608* |  | S_069 | 0.715* | -0.172* |  | 0.511* |
| F_070 | 0.258* | 0.400* |  | 0.535* |  | S_070 | 0.755* | -0.283* |  | 0.409* |
| F_071 | -0.022 | 0.813* |  | 0.788* |  | S_071 | 0.754* | 0.052 |  | 0.871* |
| F_072 | 0.145* | 0.629* |  | 0.695* |  | S_072 | 0.636* | 0.063 |  | 0.775* |
| F_073 | -0.002 | 0.849* |  | 0.856* |  | S_073 | 0.833* | -0.002 |  | 0.845* |
| F_90A | 0.795* | 0.085 | 0.778* |  |  | S_90A | 0.404* | 0.516* | 0.873* |  |
| F_90B | 0.858* | 0 | 0.783* |  |  | S_90B | 0.356* | 0.613* | 0.895* |  |
| F_091 | 0.542* | 0.389* | 0.727* |  |  | S_091 | 0.491* | 0.443* | 0.849* |  |
| F_092 | 0.580* | 0.341* | 0.783* |  |  | S_092 | 0.335* | 0.591* | 0.878* |  |
| F_093 | 0.426* | 0.167* | 0.57* |  |  | S_093 | 0.198* | 0.660* | 0.812* |  |
| F_094 | 0.655* | -0.031 | 0.837* |  |  | S_094 | -0.006 | 0.884* | 0.921* |  |
| F_095 | 0.553* | 0.033 | 0.832* |  |  | S_095 | -0.046 | 0.899* | 0.875* |  |
| F_096 | 0.228* | 0.535* |  | 0.703* |  | S_096 | 0.623* | 0.194* |  | 0.883* |
| F_104 | 0.517* | -0.141* |  |  |  |  |  |  |  |  |
|  | CORR | 0.349 | CORR | 0.419 |  |  | CORR | 0.588 | CORR | 0.649 |

**Table S-13. REALS Residential Maintenance scales, proxy report factor analysis**

| **Proxy Residential Frequency** | | | | |  | **Proxy Residential Support** | | | | |
| --- | --- | --- | --- | --- | --- | --- | --- | --- | --- | --- |
|  | EFA | | CFA | |  |  | EFA | | CFA | |
|  | F1 | F2 | F1 | F2 |  |  | F1 | F2 | F1 | F2 |
| F_060 |  | 0.812* |  | 0.827* |  | S_060 |  | 0.887* |  | 0.893* |
| F_076 | 0.96* |  | 0.841* |  |  | S_076 | 0.914* |  | 0.863* |  |
| F_077 | 0.818* |  | 0.741* |  |  | S_077 | 0.772* |  | 0.713* |  |
| F_078 | 0.842* |  | 0.856* |  |  | S_078 | 0.875* |  | 0.919* |  |
| F_079 |  |  |  |  |  | S_079 |  |  |  |  |
| F_080 | 0.442* |  | 0.791* |  |  | S_080 | 0.595* |  | 0.853* |  |
| F_083 |  | 0.764* |  | 0.874* |  | S_083 |  | 0.751* |  | 0.883* |
| F_084 |  | 0.746* |  | 0.879* |  | S_084 |  | 0.788* |  | 0.926* |
| F_085 |  | 0.75* |  | 0.862* |  | S_085 |  | 0.833* |  | 0.891* |
| F_086 |  | 0.812* |  | 0.946* |  | S_086 |  | 0.893* |  | 0.915* |
| F_087 |  | 0.887* |  | 0.938* |  | S_087 |  | 0.926* |  | 0.938* |
| F_101 |  |  |  |  |  | S_101 |  |  |  |  |
| F_103 |  |  |  |  |  | S_103 |  |  |  |  |
|  | CORR | 0.484 | CORR | 0.604 |  |  | CORR | 0.614 | CORR | 0.73 |

**Table S-14. REALS Work/School Performance scale, proxy report factor analysis**

| **Work/School Performance Frequency** | | |  | **Work/School Performance Support** | | |
| --- | --- | --- | --- | --- | --- | --- |
|  | EFA | CFA |  |  | EFA | CFA |
|  | F1 | F1 |  |  | F1 | F1 |
| F_050 | 0.688* | 0.747* |  | S_050 | 0.785* | 0.906* |
| F_052 | 0.658* | 0.683* |  | S_052 | 0.703* | 0.832* |
| F_053 | 0.781* | 0.776* |  | S_053 | 0.909* | 0.852* |
| F_054 | 0.744* | 0.846* |  | S_054 | 0.879* | 0.861* |
| F_055 | 0.824* | 0.768* |  | S_055 | 0.860* | 0.826* |
| F_056 | 0.754* | 0.691* |  | S_056 | 0.868* | 0.868* |

**Table S-15. REALS Work/School Readiness scale, proxy report factor analysis**

| **Work/School Readiness Frequency** | | |  | **Work/School Readiness Support** | | |
| --- | --- | --- | --- | --- | --- | --- |
|  | EFA | CFA |  |  | EFA | CFA |
|  | F1 | F1 |  |  | F1 | F1 |
| F_027 | 0.968* | 0.975* |  | S_027 | 0.969* | 0.968* |
| F_028 | 0.994* | 0.947* |  | S_028 | 0.930* | 0.971* |
| F_029 | 0.800* | 0.761* |  | S_029 | 0.779* | 0.786* |
| F_030 | 0.782* | 0.698* |  | S_030 | 0.738* | 0.81* |
| F_048 | 0.966* | 0.953* |  | S_048 | 0.910* | 0.95* |
| F_100 | 0.922* | 0.909* |  | S_100 | 0.804* | 0.934* |
| F_147 | 0.929* | 0.956* |  | S_147 | 0.926* | 0.937* |
